# The Global Diversity of Hemichordata

**DOI:** 10.1371/journal.pone.0162564

**Published:** 2016-10-04

**Authors:** Michael G. Tassia, Johanna T. Cannon, Charlotte E. Konikoff, Noa Shenkar, Kenneth M. Halanych, Billie J. Swalla

**Affiliations:** 1 Department of Biology, University of Washington, Seattle, WA, United States of America; 2 Friday Harbor Laboratories, University of Washington, Friday Harbor, WA, United States of America; 3 Department of Biological Sciences, Auburn University, Auburn, AL, 36849, United States of America; 4 Department of Zoology, Naturhistoriska riksmuseet, Stockholm, SE-104 05, Sweden; 5 Department of Zoology, George S. Wise Faculty of Life Science, Tel-Aviv University, Tel-Aviv, Israel; NORWAY

## Abstract

Phylum Hemichordata, composed of worm-like Enteropneusta and colonial Pterobranchia, has been reported to only contain about 100 species. However, recent studies of hemichordate phylogeny and taxonomy suggest the species number has been largely underestimated. One issue is that species must be described by experts, and historically few taxonomists have studied this group of marine invertebrates. Despite this previous lack of coverage, interest in hemichordates has piqued in the past couple of decades, as they are critical to understanding the evolution of chordates–as acorn worms likely resemble the deuterostome ancestor more closely than any other extant animal. This review provides an overview of our current knowledge of hemichordates, focusing specifically on their global biodiversity, geographic distribution, and taxonomy. Using information available in the World Register of Marine Species and published literature, we assembled a list of 130 described, extant species. The majority (83%) of these species are enteropneusts, and more taxonomic descriptions are forthcoming. Ptychoderidae contained the greatest number of species (41 species), closely followed by Harrimaniidae (40 species), of the recognized hemichordate families. Hemichordates are found throughout the world’s oceans, with the highest reported numbers by regions with marine labs and diligent taxonomic efforts (e.g. North Pacific and North Atlantic). Pterobranchs are abundant in Antarctica, but have also been found at lower latitudes. We consider this a baseline report and expect new species of Hemichordata will continue to be discovered and described as new marine habitats are characterized and explored.

## Introduction

Hemichordata is a phylum of marine invertebrates occupying an extensive range of ocean depths and habitats. “Hemichordata” Bateson, 1885 comes from the Greek prefix *hemi* (“half”) and the Latin root *chorda* (“cord”) [1]. The oldest available description of a hemichordate dates back to 1825, when Eschscholtz first described *Ptychodera flava* [2]. The hemichordate fossil record consists primarily of graptolite pterobranchs that were diverse by the mid-Cambrian [3]. In addition, a large and well-preserved fossil pterobranch recently discovered in China provides evidence of this phylum’s presence in the early Cambrian [4]. By comparison, a well-developed enteropneust, *Spartobranchus tenuis*, is known from the mid-Cambrian [5] and has been proposed to closely ally with modern torquaratorid acorn-worms [6]. Individuals of this species are described to have dwelled within a secreted fibrous tube. As enteropneusts likely resemble the deuterostome ancestor [7–11], investigation into this phylum’s fossil record is not only important for understanding hemichordate ancestral form, but also to the morphology of the last common ancestor to the deuterostomes.

**Fig 1:**
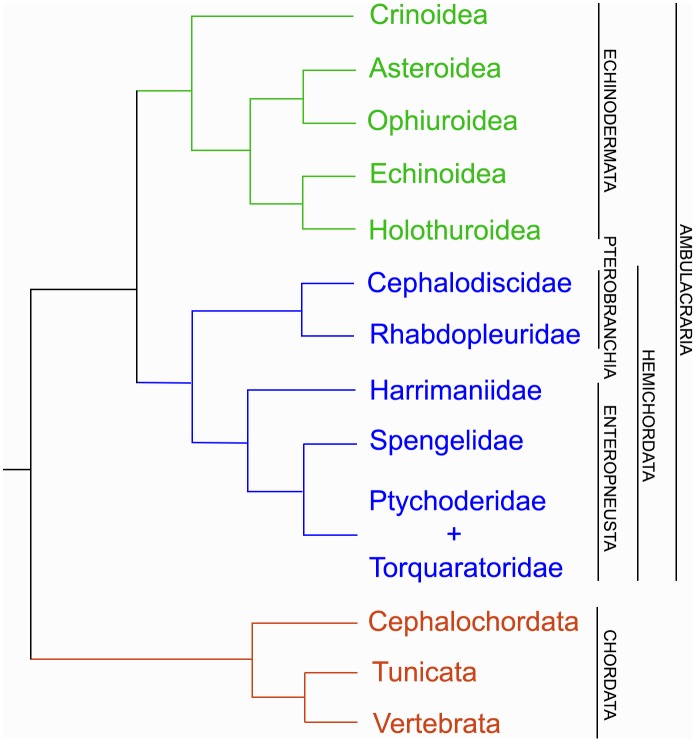
Deuterostome Phylogeny. Consensus relationships among deuterostome taxa are shown. Current data provides high-support for Classes Pterobranchia and Enteropneusta as reciprocally monophyletic. In addition, phylogenomic evidence suggests the enteropneust family, Torquaratoridae, fall within the Ptychoderidae. This tree utilizes consolidated data from 16S +18S rRNA, and phylogenomic studies from multiple sources [15, 27, 28, 100].

Hemichordates and Echinoderms form a monophyletic group called Ambulacraria [11, 12, 13]. Together, possibly with Xenoturbellida, these phyla are sister taxa to chordates (Fig 1) [14, 15]. Historically, hemichordates were classified as chordates due to the presence of gill slits in both hemichordates and chordates. They were, however, later placed in their own phylum [1, 16]. As the name suggests, hemichordates share many of the hallmark characteristics of chordates. Enteropneust hemichordates possess gill-slits, a larval post-anal tail (observed in members of the Harrimaniidae), a dorsal hollow nerve-cord, and *Hox-*specified antero-posterior body axis [9, 17–19]. In addition, the hemichordate stomochord, a structure projecting anteriorly into the proboscis, has been investigated as a possible homologue of the chordate notochord [1, 16, 20–22]. Due to these similarities, hemichordates have immense potential for studying origins of chordates and evolution of deuterostome phyla [7–11]. For example, gene expression studies in hemichordates can provide clues on the developmental origins of gill slits and chordate nervous system [7–9, 18–19, 23].

**Fig 2:**
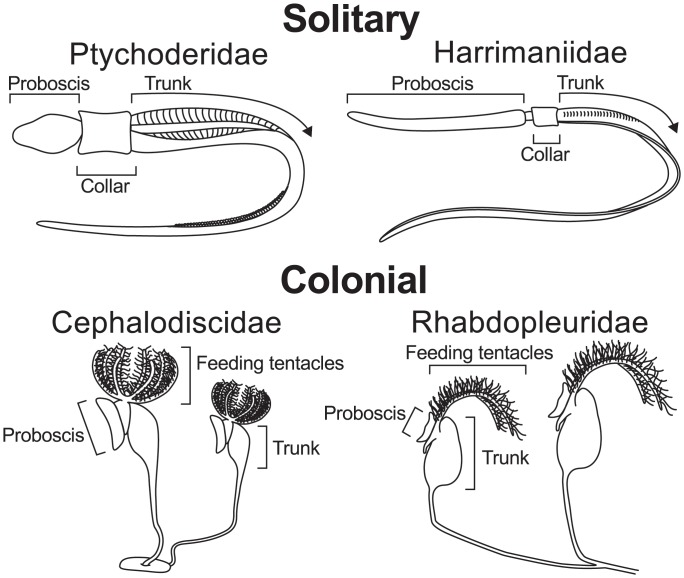
Body Plans of Hemichordate Species present Throughout the World. Commonly studied free-living acorn worms (enteropneusts) include members of the A) Ptychoderidae and B) Harrimaniidae. Enteropneusts have most often been found in coastal areas in shallow and deep waters. In contrast, extant pterobranchs C) Cephalodiscidae and D) Rhabdopleuridae often inhabit the deep sea and southern polar regions, but some also occur in warm shallow water. Pterobranch species are colonial and individuals are connected to each other via long, branched stalks. Redrawn from Rychel and Swalla (2009) [65].

**Fig 3:**
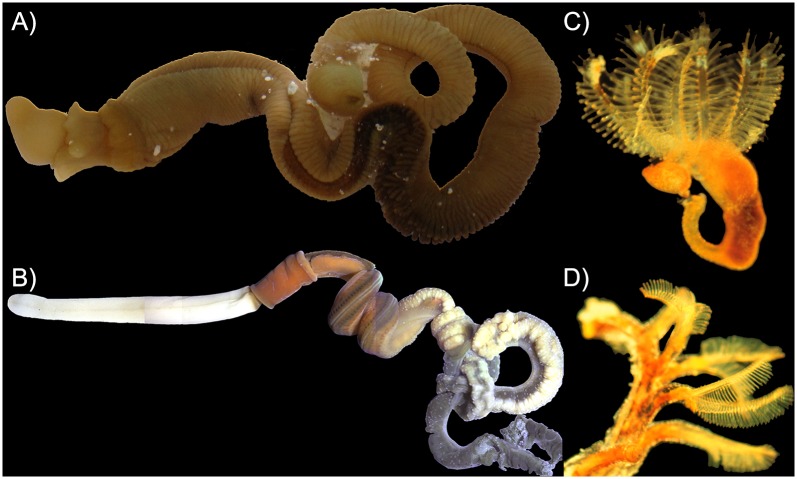
Images of Hemichordate Species found around the World. A) *Ptychodera flava* (the Hawaiian acorn worm), has been found in many different marine ecoregions of the world. It was the first described hemichordate species [2]. B) *Saccoglossus bromophenolosus* has been found in the waters of Maine and Washington state. This species was apparently introduced from Maine to Washington due to the oyster industry in the early 1900s [45]. C) Zooid of the pterobranch *Cephalodiscus gracilis*, a species found in Bermuda. D) *Rhabdopleura normani* zooids living within a coenecium. Images: A) and B) photo credit Billie J. Swalla, C) and D) photo credit Kenneth M. Halanych.

Hemichordates are currently organized into two extant classes: Enteropneusta (Gegenbaur, 1870) and Pterobranchia (Lankester, 1877). Although previous studies have suggested enteropneusts may be paraphyletic, with pterobranchs arising from within them [6, 11, 12, 24–26], recent findings have provided strong support for Enteropneusta and Pterobranchia as reciprocally monophyletic (Fig 1) [27, 28]. Extant hemichordates exhibit two distinct body plans: solitary acorn worms (Class Enteropneusta) and sessile, colonial filter-feeders (Class Pterobranchia) (Figs 2 and 3). Within the last decade, family relationships within Enteropneusta have undergone revision, whereas groups within Pterobranchia, *Rhabdopleura* and *Cephalodiscus*, have remained consistent [27, 28]. Enteropneusts were thought to be comprised of four monophyletic families: Harrimaniidae, Spengelidae, Ptychoderidae, and Torquaratoridae. However, recent phylogenomic evidence provides strong support for placement of the deep-sea torquaratorids within Ptychoderidae [28].

Adult enteropneusts have a tripartite body plan exhibiting a proboscis, collar, and trunk along the anterior-to-posterior axis (Fig 2). The proboscis can vary in length among different species and facilitates burrowing [30]. In some species, the proboscis may be quite short and resembles an acorn, hence the group’s common name “acorn worms”. The heart-kidney complex (sometimes referred to as the atrial complex) resides in the lumen of the posterior proboscis, near the collar, and is supported by the stomochord [16]. The collar contains the dorsal neural-tube, as well as an extensive epidermal nerve network that extends through the rest of the animal [9, 17, 31]. The mouth is located ventrally in between the collar and proboscis. The trunk contains gonads and viscera, terminating posteriorly with the anus in adults. However, a post-anal tail, considered by many to be a chordate character, is present in juvenile harrimaniid enteropneusts [22]. Gill slits can be seen extending along the length of the trunk. Enteropneusts may have many pairs of gill slits, while pterobranchs have one pair (*Cephalodiscus*) or none (*Rhabdopleura*) [16, 32].

Though hemichordates are infrequently collected in surveys, enteropneusts are more often found than pterobranchs and the number of accepted enteropneust species continues to grow [33]. Enteropneusts inhabit benthic substrates from the intertidal to the deep sea [24, 34–36]. In soft-sediment intertidal habitats, their coiled, rope-like fecal casts may be exposed above their burrows. For several deep-sea species, research has been done on their characteristic sediment-surface spiral, feeding traces [37]. Although species like *Ptychodera flava* may measure >5cm, the miniaturized meiofaunal enteropneust *Meioglossus psammophilus* grows to a mere 0.6mm as an adult [38], and *Balanoglossus gigas* (Müller in Spengel 1893) has been observed to grow to 2.5m in length [39, 40]. The majority of burrowing species are deposit-feeders, utilizing mucous and cilia to capture and transport food particles to the mouth. Other species are suspension-feeders and use ciliary currents to capture food particles within burrows they have carved out of mud or sand [41, 42]. Deep-sea epifaunal species appear to directly deposit feed with their mouth (KMH, personal observation).

There are currently only two hemichordate monographs (Spengel 1893 [43], Van der Horst 1939 [44]), unfortunately, these works are outdated, and cover only enteropneusts. Morphological features such as proboscis length, muscle fiber arrangement, and gill slit shape may be used to distinguish among species [43, 44]. Additionally, the number of pharyngeal gill slits may distinguish species, but it should be noted number can vary with age [43–45]. Although most enteropneusts reproduce sexually via broadcast spawning [16], brooding has also been described [26]. Embryos develop into planktonic feeding-larvae called “tornaria” in Ptychoderidae and Spengelidae; in contrast, members of the Family Harrimaniidae exhibit direct development [19, 46–49]. Data on development of torquaratorid acorn-worms is still under investigation. These animals have also been shown to possess the capacity for extensive regeneration–reconstructing entire proboscis and collar structures from fragments of the animal’s trunk [50, 51].

In contrast to enteropneusts, remaining species of hemichordates belong to the colonial Pterobranchia [52] (Fig 3C and 3D). Classically, pterobranchs and graptolites were placed within separate clades [53, 54]; however, recent fossil evidence indicates pterobranchs as closely related to graptolites, and thus the two clades have been combined [4, 30, 55–58]. Fewer than 30 extant pterobranch species have been described [59]. Though graptolites display expansive evolutionary radiation [55, 56, 60], all extant pterobranch species belong to one of two genera: *Rhabdopleura* or *Cephalodiscus*. Note that the previously recognized genera (*Atubaria*) is not considered valid because the only description of its sole member, *Atubaria heterolopha*, [61] is regarded as a questionable species [62]. Modern pterobranchs have been described to reside on shells and hard substrates in shallow water and deeper species have been observed to build colonies up to several centimeters in size [63, 64].

Pterobranchs are colonial [65], whereas all extant enteropneusts are solitary. Adults pterobranchs superficially resemble hydroids or bryozoans, and were originally misidentified as such [66, 67]. Pterobranch zooids are connected via a stalk with each individual zooid displaying the hemichordate body plan: proboscis, collar, and trunk [50]. The shield-shaped proboscis of each zooid is used to secrete material that will harden into a tube, called a ‘coenecium’, and ultimately houses the adult pterobranch [16, 30]. Zooids of each species may or may not contain gill slits (*Cephalodiscus* species have one pair, but *Rhabdopleura* species have none). Collars of pterobranchs possess a feeding structure similar to a lophophore, a filter feeding structure comprised of ciliated arms with tentacles [32, 62, 68] (Fig 3). Akin to enteropneusts, the pterobranch mouth is located between the collar and trunk; however, pterobranchs possess a U-shaped gut (as opposed to the through-gut of acorn worms). Colonies are comprised of both male and female zooids [69, 70]. Fertilization occurs internally, development occurs within the coenecium, and then the swimming planula-like larvae are released [69, 71]. In addition, colony expansion occurs though asexual budding [69]. Pterobranchs are small and easy to overlook, thus they have not been extensively studied [54, 69, 71].

### Hemichordate taxonomy

Hemichordates have been the focus of recent research efforts due to their immense potential to elucidate deuterostome evolution and development, particularly in the context of chordate origins. Developmental and genetic evidence suggest that both deuterostome and chordate ancestors were worm-like organisms, resembling a solitary enteropneust [9, 24]. Current taxonomy places 130 described hemichordate species into 24 different genera. Over the last three decades, hemichordate taxonomy and phylogenetics have received greater scientific interest [6, 26, 27, 35, 38, 45, 72–77]. Recent taxonomic work includes new molecular systematics [24, 27, 28, 78] and species descriptions [26, 27, 35, 38, 79], in addition to more taxonomic manuscripts in preparation [33].

Some hemichordate genera are debatable, as they are composed of a single described species. For example, *Planctosphaera* may simply represent the tornaria larva of an undescribed deep-sea enteropneust [61, 80, 81]. These groups have been excluded from our inventory, as their status as valid species is not clear.

## Methods

### Hemichordate species global inventory and validation

We compiled a list of species using the World Register of Marine Species (WoRMS; http://www.marinespecies.org) [59], which links to the Hemichordata World Database [82] and crossed checked species with the published literature. The Catalogue of Life 2011 Annual Checklist [83] and Global Biodiversity Information Facility (http://www.gbif.org/ accessed May, 2014) were used for validation, and synonymous species names were consolidated. For consistency, hemichordates are referred to by the genus and species names displayed in the WoRMS database. Original species descriptions were verified via searching original reports, manuscripts and monographs. Some hemichordate genera are debatable, and several are composed of a single described species. For example, *Planctosphaera* may simply represent the tornaria larva of an undescribed deep-sea enteropneust [61, 80, 81]. See S1 Table for the complete list. Databases were last accessed in March 2014.

### Geographic distributions and biodiversity

The Ocean Biogeographic Information System [84] and SeaLifeBase [85] were utilized in addition to data obtained from primary literature. These databases compile datasets from records including government reports, museum collections, and ecological surveys; thus they are advantageous to include in these studies. Additional data sources included the Smithsonian’s National Museum of Natural History Invertebrate Zoology collection [86] and National Oceanic and Atmospheric Administration records [87].

Only publications and reports identifying hemichordates to species level were included in our assessment of global biodiversity. Notably, many database records contained hemichordates not identified to species level. Thus, our assessment is conservative and underestimates the true distribution and global biodiversity of this phylum [33]. We separated Torquaratoridae and Ptychoderidae in our assessment, though phylogenomic evidence supports the Torquaratoridae belonging within the Ptychoderidae [28]. Data collected from literature and databases were binned species into Marine Ecoregions of the World (MEOWs) and marine provinces, as given in Spalding et al. 2007 [88].

## Results

**Fig 4:**
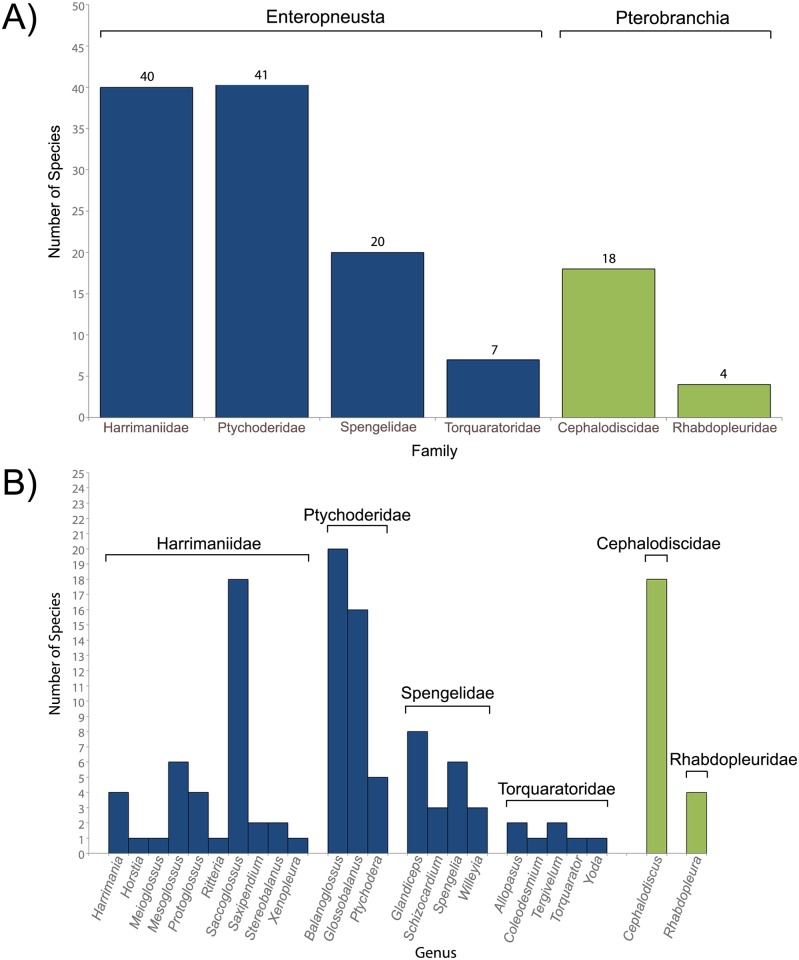
Extant Hemichordate Species belonging to Enteropneusta or Pterobranchia. The number of species within each A) family and B) each genus are shown. Note that recent taxonomic revisions have placed some species that were formerly the sole members of their own families into other families. Torquaratoridae and Ptychoderidae are shown as separate families to depict diversity within these groups; however, data support Torquaratoridae falling with Ptychoderidae [28]. See S1 Table for a comprehensive list of valid hemichordate species.

We assembled a list of extant species and localities in an effort to gain insight into global biodiversity of hemichordates. We compiled a list of 130 described, extant species (S1 Table). Of these, 108 (83%) belong to Class Enteropneusta, while 22 (17%) are members of Pterobranchia. Distributions of hemichordate species within each family are shown in Fig 4a. Most species are placed in Ptychoderidae (41) and Harrimaniidae (40) with *Balanoglossus* containing the most species overall (20), followed by *Saccoglossus* (18) and the pterobranch genus *Cephalodiscus* (18) (Fig 4b). Note that some species currently are, or were previously, classified as sole members of their own families.

### Rate of discovery

**Fig 5:**
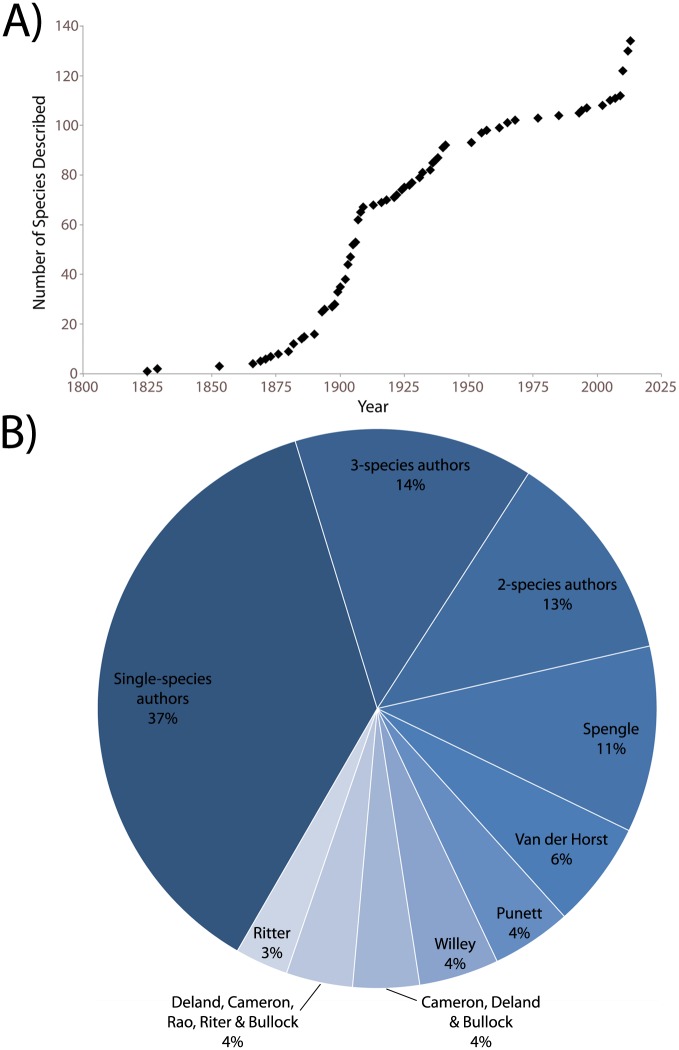
A Timeline of Hemichordate Species Discovery. A) The cumulative number of new enteropneust and pterobranch species descriptions per year is shown. B) The percentage of species described according to author. For example, ‘single-species authors’ indicates 37% of species were described by authors whom described only a single hemichordate in his/her career. Reports by Ritter alone (4) are not binned with Cameron et al. descriptions (e.g. [101] and [77], respectively). Only extant species are included.

We next examined the rate of new species discovery for extant hemichordates (Fig 5A). The author and year of discovery for each species was determined via searching original reports, such as in manuscripts or monographs. Since the first discovery of a hemichordate by Eschscholtz in 1825 [2], the rate of new species discovery has varied. Between 1893 and 1908 there were 48 new species reported. Historically, this time period corresponds to an intensive time of exploration, including the publication of Johann Wilhelm Spengel’s monograph on the Siboga expedition and Arthur Willey’s results of his expedition to the Pacific [43, 89]. In 1893 and 1907, Spengel described seven and six novel species, respectively. Arthur Willey described 6 additional species during this time (the majority reported in 1899), resulting from his expedition to the Pacific. In 1903 Punnett also published descriptions of five new species [90]. The years with the greatest numbers of new species descriptions (Fig 5A) are 1893 & 2012 (8), 1907 (9) and 2010 (10).

We found the majority of reported species (67%) were described by invertebrate taxonomists who named fewer than five hemichordate species during their lifetimes. Overall, 37% of currently described species were described by an author who would not describe any other hemichordates (Fig 5B). Spengel named the most species (14), the majority of which he described in 1893 and 1907. The most recently described species where originally discovered by Bullock and colleagues and the taxonomy was completed by Christopher B. Cameron and colleagues [35, 77], who collectively described ten of these species in 2010. Also, recent work on deep-sea taxa have revealed many unknown enteropneust species, with many more yet to be described [25, 36, 91]. As deep-sea sample collection becomes increasingly accessible, many hemichordate species new to science are rapidly being discovered and described [6, 24, 25, 27, 29, 34, 36, 37]. The next 10–20 years will be important for discovery and publishing new hemichordate species.

### Global biodiversity

**Fig 6:**
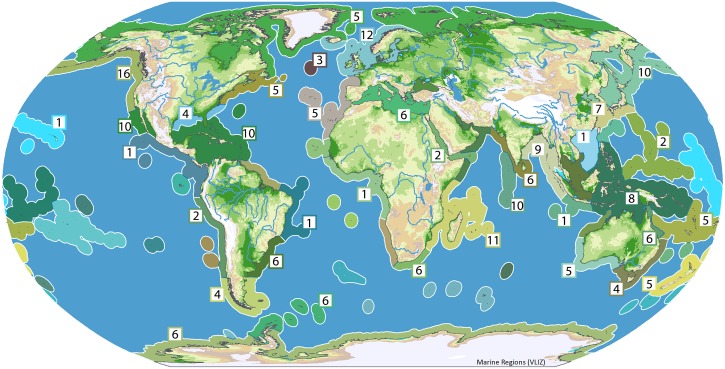
Biogeographical Distribution of Enteropneust and Pterobranch species. Depiction of the number of unique species reported in each geographic region. Geographic regions are adapted from the marine provinces of Spalding et al. 2007 [88]. These numbers are an underestimation of true species diversity, as there are manuscripts *in preparation* and many described specimens [33]. See S1 Table for detailed marine province information. Map image: Courtesy of VLIMAR [102]. Figure modified from source material in reference [88]. Original figure: http://www.marineregions.org/gazetteer.php?p=image&pic=64936.

Analysis of the 232 Marine Ecoregions of the World (MEOWs) [88] revealed the majority of reports for described species were collected from intertidal zones on coasts or in shallow water, which are most accessible marine habitats. We found reports of hemichordates throughout the world’s oceans (Fig 6). Hemichordate species were reported in 97 different MEOWs (41.8% of total MEOWs). In addition to these MEOWs, 3 species have been described from the deep sea in the North Atlantic [25]. This new MEOW has been added to our inventory (S2 and S3 Tables). *Ptychodera flava* was found in the most MEOWs (16), its habitat including many coastal areas of the Indian and Pacific Oceans, but the population boundaries of this species need to be accurately accessed. *Rhabdopleura normani* is also reported from many MEOWs (9), from the tropical waters of Bermuda to the temperate North Atlantic and Southern Ocean, but several of these reports likely represent unrecognized species (KMH, unpublished data). Other species, such as *Cephalodiscus sibogae*, have only been reported once and have not been seen again [92]. Overall there were 40 MEOWs associated with a report of only one species.

We also examined global species diversity in the context of marine provinces. The majority of described species have been found living in Northern Atlantic and Pacific waters, as well as the Western Indian Ocean. Seven marine provinces were found to have 10 or more species inhabiting them. These provinces are the Cold Temperate NE Pacific, Northern European Seas, Tropical NW Atlantic, Western Indian Ocean, Cold Temperate NW Pacific, Warm Temperate NE Pacific, and Central Indian Ocean Islands (S1 Table). Enteropneusts comprised the majority of the inhabitant hemichordates.

The Cold Temperate Northeast Pacific province includes the Pacific Northwest and parts of California. Interestingly, there is high species diversity along the entirety of the California coast (16 species) and in the Pacific Northwest of the United States (10 species), where many marine labs are located (Fig 6). Many species have been documented in Northern European Seas (e.g. Celtic Seas, etc.), two of which are pterobranchs. The Western Indian Ocean also hosts several described hemichordates, many of which were initially discovered on expeditions occurring in the late 1800s and early 1900s. This province includes Delagoa and Madagascar. The biodiversity of hemichordates has also been well documented in the Tropical Northwestern Atlantic (11 species, 4 of which are pterobranchs). This province includes Bermuda, the Bahamas, the Caribbean and parts of the Gulf of Mexico. Seven species (all of which are enteropneusts) have been described in the waters surrounding Brazil. The Brazilian coast is surrounded by three distinct marine provinces: the North Brazil Shelf, Tropical Southwestern Atlantic and Warm Temperate Southwestern Atlantic.

At least 4 enteropneust species have been recently discovered around Antarctica [6] but have yet to be described. As this discovery has only made recently, future Southern Ocean expeditions may yield interesting results for enteropneust biodiversity. Unlike acorn worms, pterobranch species distribution is largely localized to the high latitudes of the Southern Hemisphere. Many pterobranch species have been found in the Scotia Sea (6 species), Antarctic (6 species), and Magellanic (4 species) marine provinces. Note, however, that pterobranchs have also been found living in warmer waters, including Bermuda and the Azores (S3 Table). It will be interesting to investigate the reasons behind the biased distribution of pterobranch species to the Southern Ocean regions.

There are currently 18 marine provinces with no described species (29% of provinces delineated in Spalding et al. 2007 [88]). See S1 Table for a complete list of hemichordate species belonging to each marine province. Further, 8 (13%) marine provinces currently only have a report of one species. Provinces where only one species has been reported include the Gulf of Guinea, the South China Sea and Hawaii [43, 84, 93]. This is likely due to sampling efforts, as the vast majority of reports listed species found in intertidal zones along coasts of areas with a significant human population. Future studies aimed at exploring the marine biodiversity of lesser explored marine environments, as well as the deep sea, may yield new and useful information providing us with a better picture of the true biodiversity of this phylum.

## Discussion

Hemichordates have been found throughout the world’s oceans. Although our inventory of hemichordate biodiversity is an underestimation of the true global diversity [33], patterns in their distribution have begun to emerge. Regions where there have been large taxonomic efforts indeed display the highest numbers of diversity (among these are the NE Pacific, Tropical Western Atlantic, and Northern European Seas). On the other hand, regions such as the Brazilian shelf, have only one described species of hemichordate, despite displaying otherwise high levels of biodiversity (Fig 6) [94]. Though specimens may have been collected in these regions, understanding the diversity and distribution of hemichordates is directly contingent upon taxonomic efforts. Taxonomic identification of specimens collected from regions such as the Brazilian Shelf, Eastern Indo-Pacific, and Tropical Eastern Pacific will be important to our understanding of hemichordate biodiversity.

Advances in molecular and underwater-imaging methods have provided access to specimens previously unattainable by classic collection techniques. Recently, several novel deep-sea enteropneust species have been discovered using ROV-mounted cameras, molecular systematics, or a combination of both [24, 27, 29, 34, 36, 37, 79, 95]. These techniques have facilitated powerful sampling methods for future ecological, behavioral, and biogeographic studies. Furthermore, genetic methods may also be used for phylogeographic analyses for species of widespread distribution. *Ptychodera flava* and *Rhabdopleura normani*, the two hemichordate species with the highest reported species distribution, will benefit highly from these studies.

As hemichordate species continue to be discovered and described (Fig 5A), our understanding of these animals’ biology will continue to grow. Future hemichordate research will greatly benefit a broad variety of fields, including evolution, development and regeneration, in addition to systematics and taxonomy. Hemichordates occupy an important position on the tree of life [11, 96, 97], and provide particular insight into body patterning and deuterostome origins. For instance, recent neural gene expression studies have suggested a common origin among bilaterian central nervous systems [17, 31, 98, 99]. Furthermore, insight into origins of the chordate notochord may benefit from studying the hemichordate stomochord. Studying this group of animals may also benefit human health, as some species (such as *Ptychodera flava*) are capable of regenerating anterior and posterior adult body structures after complete amputation, including the heart-kidney complex and stomochord [50, 51].

Our species inventory is certainly incomplete, as many new species are still being described. Our analysis underestimates the biodiversity; this is a result of the many hemichordates that are not identifiable to a species level [33]. In addition, we have also excluded reports of species’ locales that were not specific enough to be categorized into at least one MEOW province. Regarding taxonomy, re-examining species with wide geographic ranges will be valuable to determine if they constitute multiple species.

## Supporting Information

S1 Table Extant Hemichordate Species and their Biogeographic Distribution. (PDF)

S2 Table Extant Hemichordate Species and Geographic Regions They Inhabit. (PDF)

S3 Table Number of Extant Hemichordate Species in the Context of Marine Provinces. (PDF)
