# Supplementary material for: The Global Diversity of Hemichordata
Source: PLoS One. 2016 Oct 4;11(10):e0162564. doi: 10.1371/journal.pone.0162564 (PMC5049775; doi:10.1371/journal.pone.0162564)
Supplement: S1 Table — (PDF) [file pone.0162564.s001.pdf]

S1 Table. Extant Hemichordate Species and their Biogeographic Distribution. Discovery and marine province information are shown for each species. Information regarding specific Marine Ecoregions of the World (MEOWs) [1] each species inhabits is available via the World Online Register of Marine Species (WoRMS). * The "Deep Sea North Atlantic" province was added to the list provided by Spalding et al. (2007) due to species inhabiting areas outside of previously described MEOWs.

Family	Species	Discoverer and Date	Marine Provinces	Ref.
**Harrimaniidae**	*Harrimania borealis*	Okuda & Yamada, 1955 [2]	Cold Temperate NW Pacific	[2]
	Harrimania kupfferi	von Willemoes-Suhm, 1871 [3]	Northern European Seas, Arctic	[3-7]
	Harrimania maculosa	Ritter, 1900 [8]	Cold Temperate NE Pacific, Arctic	[8, 9]
	Harrimania planktophilus	Cameron, 2002 [10]	Cold Temperate NE Pacific	[10]
	Horstia kincaidi	Deland, Cameron, Rao, Ritter & Bullock, 2010 [9]	Cold Temperate NE Pacific	[9]
	Meioglossus psammophilus	Worsaae, Sterrer, Kaul-Strehlow, Hay-Schmidt & Giribet, 2012 [11]	Tropical NW Atlantic	[11]
	Mesoglossus bournei	Menon, 1904 [12]	Bay of Bengal	[12, 13]
	Mesoglossus caraibicus	van der Horst, 1924 [14]	Tropical NW Atlantic	[6, 14]
	Mesoglossus gurneyi	Robinson, 1927 [15]	Mediterranean Sea, Red Sea	[15-18]
	Mesoglossus intermedius	Deland, Cameron, Rao, Ritter & Bullock, 2010 [9]	Cold Temperate NE Pacific	[9]
	Mesoglossus macginitiei	Deland, Cameron, Rao, Ritter & Bullock, 2010 [9]	Cold Temperate NE Pacific	[9]
	Mesoglossus pygmaeus	Hinrichs & Jacobi, 1938 [19]	Northern European Seas	[6, 16, 19, 20]
	Protoglossus bocki	Cedhagen & Hansson, 2012 [21]	Northern European Seas	[21]
	Protoglossus graveolens	Giray & King, 1996 [22]	Cold Temperate NW Atlantic	[4, 6, 22]
	Protoglossus koehleri	Caullery & Mesnil, 1900 [23]	Northern European Seas	[4, 6, 23, 24]
	Protoglossus mackiei	Deland, Cameron, Rao, Ritter & Bullock, 2010 [9]	Cold Temperate NE Pacific	[9]
	Ritteria ambigua	Deland, Cameron, Rao, Ritter &	Cold Temperate NE Pacific	[9]

		Bullock, 2010 [9]		
	Saccoglossus apatensis	Thomas, 1955 [25]	SW Australian Shelf	[4, 25]
	Saccoglossus aulakoeis	Thomas, 1968 [26]	SW Australian Shelf	[26]
	Saccoglossus bromophenolosus	King, Giray & Kornfield, 1994 [27]	Cold Temperate NE Pacific, Cold Temperate NW Atlantic	[4, 6, 9, 27]
	Saccoglossus horsti	Brambell & Goodhart, 1941 [28]	Northern European Seas	[4, 6, 28]
	Saccoglossus hwangtauensis	Tchang and Koo, 1935 [29]	Cold Temperate NW Pacific, Warm Temperate NW Pacific	[16, 29]
	Saccoglossus inhacensis	Kapelus, 1936 [30]	Western Indian Ocean, Agulhas	[30, 31]
	Saccoglossus kowalevskii	Agassiz, 1873 [32]	Cold Temperate NW Atlantic, Warm Temperate NW Atlantic	[4, 6, 32]
	Saccoglossus madrasensis	Rao, 1957 [33]	West & South Indian Shelf, Bay of Bengal	[13, 33]
	Saccoglossus mereschkowskii	Wagner, 1885 [34]	Arctic	[6, 31, 34, 35]
	Saccoglossus otagoensis	Benham, 1899 [36]	SW Australian Shelf, Southern New Zealand	[26, 36-38]
	Saccoglossus palmeri	Cameron, Deland & Bullock, 2010 [39]	Cold Temperate NE Pacific	[39]
	Saccoglossus porochordus	Cameron, Deland & Bullock, 2010 [39]	Warm Temperate NE Pacific	[39]
	Saccoglossus pusillus	Ritter, 1902 [40]	Cold Temperate NE Pacific, Warm Temperate NE Pacific	[9, 40]
	Saccoglossus rhabdorhyncus	Cameron, Deland & Bullock, 2010 [39]	Cold Temperate NE Pacific	[39]
	Saccoglossus ruber	Tattersall, 1905 [41]	Northern European Seas	[4, 6, 41]
	Saccoglossus shumaginensis	Cameron, Deland & Bullock, 2010 [39]	Cold Temperate NW Pacific	[39]
	Saccoglossus sonorensis	Cameron, Deland & Bullock, 2010 [39]	Warm Temperate NE Pacific	[39]
	Saccoglossus sulcatus	Spengel, 1893 [42]	Warm Temperate NW Pacific	[42]
	Saxipendium coronatum	Woodwick & Sensenbaugh, 1985 [43]	Tropical East Pacific, Galapagos	[4, 43]
	Saxipendium implicatum	Holland, Osborn & Kuhnz, 2012 [44]	Cold Temperate NE Pacific	[44]
	Stereobalanus canadensis	Spengel, 1893 [42]	Northern European Seas, Cold Temperate NW Atlantic, Warm Temperate NE Pacific	[4, 6, 9, 42]

	Stereobalanus willeyi	Ritter, 1904 [9]	Warm Temperate NE Pacific	[9]
	Xenopleura vivipara	Gilchrist, 1925 [45]	Agulhas	[45]
**Ptychoderidae**	*Balanoglossus apertus*	Spengel, 1893 [42]	Central Indian Ocean Islands, Eastern Brazil	[42, 46]
	Balanoglossus aurantiaca	Girard, 1853 [47]	Cold Temperate NW Atlantic	[4, 47, 48]
	Balanoglossus australiensis	Hill, 1894 [49]	Eastern Coral Triangle, NE Australian Shelf, East Central Australian Shelf, Southern New Zealand, Sahul Shelf	[4, 6, 38, 49, 50]
	Balanoglossus borealis	Willey, 1899 [48]	Cold Temperate NW Pacific	[48]
	Balanoglossus capensis	Gilchrist, 1908 [51]	Western Indian Ocean	[51]
	Balanoglossus carnosus	Müller in Spengel, 1893 [52]	Andaman, Central Indian Ocean Islands, Eastern Coral Triangle, Tropical SW Pacific, Warm Temperate NW Pacific, NE Australian Shelf, Sahul Shelf, Tropical NW Pacific	[2, 6, 42, 46, 48, 50, 52]
	Balanoglossus clavigerus	delle Chiaje, 1829 [53]	Lusitanian, Northern European Seas, Warm Temperate SW Atlantic, Mediterranean Sea	[4, 6, 18, 42, 53]
	Balanoglossus gigas	Müller in Spengel, 1893 [52]	Tropical NW Atlantic, Warm Temperate NW Atlantic, Warm Temperate SW Atlantic	[6, 42, 48, 54, 55]
	Balanoglossus hydrocephalus	van der Horst, 1940 [56]	Western Indian Ocean, Agulhas	[56, 57]
	Balanoglossus jamaicensis	Willey, 1899 [48]	Tropical NW Atlantic	[48]
	Balanoglossus misakiensis	Kuwano, 1902 [58]	Warm Temperate NW Pacific	[58]
	Balanoglossus natalensis	Gilchrist, 1908 [59]	Agulhas	[59, 60]
	Balanoglossus occidentalis	Ritter, 1902 [40]	Cold Temperate NE Pacific, Warm Temperate NE Pacific	[40, 61]
	Balanoglossus parvulus	Punnett, 1903 [46]	Central Indian Ocean Islands, West & South Indian Shelf	[4, 6, 46, 62]
	Balanoglossus proterogonius	Belichov, 1928 [63]	Cold Temperate NW Pacific	[63, 64]
	Balanoglossus robinii	Giard, 1882 [65]	Lusitanian	[65]

	Balanoglossus salmoneus	Giard, 1882 [65]	Lusitanian	[65]
	Balanoglossus simodensis	Miyamoto & Saito, 2007 [66]	Warm Temperate NW Pacific	[66]
	Balanoglossus stephensoni	van der Horst, 1937 [67]	Western Indian Ocean, Agulhas	[67]
	Balanoglossus studiosorum	van der Horst, 1940 [56]	Western Indian Ocean, NE Australian Shelf	[50, 56, 57]
	Glossobalanus alatus	van der Horst, 1940 [56]	Western Indian Ocean	[56, 57]
	Glossobalanus barnharti	Cameron & Ostiguy, 2013 [68]	Warm Temperate NE Pacific	[68]
	Glossobalanus berkeleyi	Willey, 1931 [69]	Cold Temperate NE Pacific	[69, 70]
	Glossobalanus crozieri	van der Horst, 1924 [14]	Tropical NW Atlantic, Warm Temperate SW Atlantic	[14, 31, 71, 72]
	Glossobalanus elongatus	Spengel, 1904 [73]	Mediterranean Sea	[73]
	Glossobalanus hartmanae	Cameron & Ostiguy, 2013 [68]	Warm Temperate NE Pacific	[68]
	Glossobalanus hedleyi	Hill, 1897 [74]	NE Australian Shelf, Tropical SW Pacific	[25, 74]
	Glossobalanus indicus	Rao, 1955 [75]	Bay of Bengal	[75]
	Glossobalanus marginatus	Meek, 1922 [76]	Northern European Seas	[4, 6, 76, 77]
	Glossobalanus minutus	Kowalevsky, 1866 [78]	Lusitanian, Andaman, Bay of Bengal, Warm Temperate SW Atlantic, Mediterranean Sea	[4, 6, 12, 13, 78, 79]
	Glossobalanus mortenseni	van der Horst, 1932 [80]	Cold Temperate NW Pacific	[80]
	Glossobalanus parvulus	Punnett, 1906 [81]	Central Indian Ocean Islands, West & South Indian Shelf	[62, 81]
	Glossobalanus polybranchioporus	Tchang & Liang, 1965 [82]	Cold Temperate NW Pacific	[82]
	Glossobalanus ruficollis	Willey, 1899 [48]	Eastern Coral Triangle, Western Coral Triangle, South China Sea, Tropical NW Pacific	[48, 83, 84]
	Glossobalanus sarniensis	Koehler, 1886 [85]	Northern European Seas	[4, 6, 85]
	Glossobalanus williami	Cameron & Ostiguy, 2013 [68]	Warm Temperate NE Pacific	[68]
	Ptychodera asymmetrica	Punnett, 1903 [46]	Central Indian Ocean Islands	[46]
	Ptychodera bahamensis	Spengel, 1893 [42]	Warm Temperate NW Atlantic, Tropical NW	[4, 6, 42]

			Atlantic	
	Ptychodera flava	Eschscholtz, 1825 [86]	Central Indian Ocean Islands, Warm Temperate SE Pacific, West & South Indian Shelf, Western Indian Ocean, Andaman, NE Australian Shelf, East Central Australian Shelf, West Central Australian Shelf, Bay of Bengal, Hawaii, Marshall Islands, Southern New Zealand, Red Sea	[4, 6, 13, 25, 38, 46, 48, 58, 79, 84, 86-89]
	Ptychodera pelsarti	Dakin, 1916 [87]	West Central Australian Shelf	[87]
	Ptychodera viridis	Punnett, 1903 [46]	Central Indian Ocean Islands, Andaman	[4, 6, 46]
**Spengelidae**	*Glandiceps abyssicola*	Spengel, 1893 [42]	Gulf of Guinea	[42]
	Glandiceps bengalensis	Rao, 1955 [75]	Bay of Bengal	[75]
	Glandiceps coromandelicus	Spengel, 1907 [90]	Bay of Bengal	[13, 90]
	Glandiceps eximius	Spengel, 1907 [90]	Warm Temperate NW Pacific	[90]
	Glandiceps hacksi	Marion, 1885 [91]	Warm Temperate NW Pacific	[42, 91]
	Glandiceps malayanus	Spengel, 1907 [90]	Java Transitional, NE Australian Shelf	[90, 92]
	Glandiceps qingdaoensis	An & Li, 2005 [93]	Cold Temperate NW Pacific	[93]
	Glandiceps talaboti	Marion, 1876 [94]	Mediterranean Sea	[18, 42, 94]
	Schizocardium brasiliense	Spengel, 1893 [42]	Warm Temperate NW Atlantic, Tropical NW Atlantic, Warm Temperate SW Atlantic	[6, 42]
	Schizocardium californicum	Cameron & Perez, 2012 [95]	Warm Temperate NE Pacific	[95]
	Schizocardium peruvianum	Spengel, 1893 [42]	Warm Temperate SE Pacific	[31, 42]
	Spengelia alba	Willey, 1899 [48]	Western Coral Triangle	[48]
	Spengelia amboinensis	Spengel, 1907 [90]	Western Coral Triangle	[90]
	Spengelia discors	Spengel, 1907 [90]	Central Indian Ocean Islands	[90]
	Spengelia maldivensis	Punnett, 1903 [46]	Central Indian Ocean Islands	[46]
	Spengelia porosa	Willey, 1898 [96]	Tropical SW Pacific, Central Indian Ocean	[4, 6, 96]

			Islands, Western Indian Ocean	
	Spengelia sibogae	Spengel, 1907 [90]	Western Coral Triangle	[90]
	Willeyia bisulcata	Punnett, 1903 [46]	Western Indian Ocean	[46]
	Willeyia delagoensis	van der Horst, 1940 [56]	Western Indian Ocean	[56, 57]
	Willeyia loya	Petersen, 1965 [97]	Tropical SW Atlantic, Warm Temperate SW Atlantic	[97]
**Torquaratoridae**	*Allapasus aurantiacus*	Holland, Kuhnz & Osborn, 2012 [98]	Cold Temperate NW Pacific	[98]
	Allapasus insidis	Priede, Osborn, Gebruk, Jones, Shale, Rogacheva & Holland, 2012 [99]	Deep Sea North Atlantic*	[99]
	Coleodesmium karaensis	Osborn, Gebruk, Rogacheva & Holland, 2013 [100]	Arctic	[100]
	Tergivelum baldwinae	Holland, Jones, Ellena, Ruhl & Smith, 2009 [101]	Cold Temperate NE Pacific	[101]
	Tergivelum cinnabarinum	Priede, Osborn, Gebruk, Jones, Shale, Rogacheva & Holland, 2012 [99]	Deep Sea North Atlantic*	[99]
	Torquarator bullocki	Holland, Clague, Gordon, Gebruk, Pawson & Vecchione, 2005 [102]	Cold Temperate NE Pacific	[102]
	Yoda purpurata	Priede, Osborn, Gebruk, Jones, Shale, Rogacheva & Holland, 2012 [99]	Deep Sea North Atlantic*	[99]
**Cephalodiscidae**	*Cephalodiscus agglutinans*	Harmer and Ridewood, 1913 [103]	Magellanic	[103]
	Cephalodiscus atlanticus	Bayer, 1962 [104]	Tropical NW Atlantic	[6, 104]
	Cephalodiscus australiensis	Johnston and Muirhead, 1951 [105]	SE Australian Shelf	[105]
	Cephalodiscus calciformis	Emig, 1977 [106]	Western Indian Ocean	[106]
	Cephalodiscus densus	Andersson, 1907 [107]	Scotia Sea, Continental High Antarctic	[6, 107-109]
	Cephalodiscus dodecalophus	M'Intosh, 1882 [110]	Magellanic	[90, 110, 111]
	Cephalodiscus evansi	Ridewood, 1918 [112]	Northern New Zealand	[38, 112]
	Cephalodiscus	John, 1931 [113]	Scotia Sea, Continental	[113]

	fumosus		High Antarctic	
	Cephalodiscus gilchristi	Ridewood, 1908 [114]	Agulhas	[114-116]
	Cephalodiscus gracilis	Harmer, 1905 [117]	Western Coral Triangle, Tropical NW Atlantic	[90, 117, 118]
	Cephalodiscus graptolitoides	Dilly, 1993 [119]	Tropical SW Pacific	[6, 119]
	Cephalodiscus hodgsoni	Ridewood, 1907 [120]	Magellanic, Scotia Sea, Continental High Antarctic	[4, 6, 108, 120, 121]
	Cephalodiscus indicus	Schepotieff, 1909 [122]	West & South Indian Shelf	[108, 122]
	Cephalodiscus kempi	John, 1932 [123]	Scotia Sea, Continental High Antarctic	[6, 123]
	Cephalodiscus levinseni	Harmer, 1905 [117]	Cold Temperate NW Pacific	[117]
	Cephalodiscus nigrescens	Lankester, 1905 [124]	Scotia Sea, Continental High Antarctic	[4, 6, 108, 119, 124]
	Cephalodiscus sibogae	Harmer, 1905 [117]	Western Coral Triangle	[117]
	Cephalodiscus solidus	Andersson, 1907 [125]	Scotia Sea, Continental High Antarctic	[4, 6, 107, 108]
**Rhabdopleuridae**	*Rhabdopleura annulata*	Norman, 1921 [126]	Northern New Zealand, Southern New Zealand, SE Australian Shelf	[4, 6, 126, 127]
	Rhabdopleura compacta	Hincks, 1880 [128]	Northern European Seas, Tropical NW Atlantic	[4, 6, 77, 128-131]
	Rhabdopleura normani	Allman, 1869 [132]	Arctic, Lusitanian, Northern European Seas, Mediterranean Sea, Magellanic, Tropical SW Pacific, Tropical NW Atlantic	[4, 6, 129, 130, 132, 133]
	Rhabdopleura striata	Schepotieff, 1909 [122]	West & South Indian Shelf	[122]

References

1. Spalding MD, Fox HE, Allen GR, Davidson N, Ferdaña ZA, Finlayson M, et al. Marine ecoregions of the world: A bioregionalization of coastal and shelf areas. BioScience. 2007;57(7): 573-583.

2. Okuda S, Yamada M. Enteropneusta of Akkeshi Bay. Publications of the Akkeshi Marine Biological Station. 1955;6: 1-7.

3. von Willemoes-Suhm R. Biologische Beobachtungen über niedere Meeresthiere. Ueber *Balanoglossus kupfferi* aus den Oeresund. Z Wiss Zool. 1871;21: 380-396.

4. GBIF. Global Biodiversity Information Facility. 2011. Ver. 1.3.1 [cited 2014 May] Available: http://www.gbif.org/.

5. Cunningham J. Tornaria and Actinotropha of the British Coasts. Nature. 1886;34(877): 361.

6. International Oceanographic Commission of UNESCO. The Ocean Biogeographic Information System. 2007 [cited 2014 May]. Available: http://iobis.org/.

7. Hansson H. NEAT (North East Atlantic Taxa): South Scandinavian marine "Aschelminth" (excl. Nemetoda 1997 [cited 2014 May]. Available: http://www.tmbl.gu.se.

8. Ritter W. *Harrimania maculosa*, a new genus and species of Enteropneusta from Alaska, with special regard to the charcter of its notochord. Proc Wash Acad Sci. 1900;2: 111-132.

9. Deland C, Cameron C, Rao K, Ritter W, Bullock T. A taxonomic revision of the family Harrimaniidae (Hemichordata: Enteropneusta) with descriptions of seven species from the Eastern Pacific. Zootaxa. 2010: 1-30.

10. Cameron CB. The anatomy, life habits, and later development of a new species of enteropneust, *Harrimania planktophilus* (Hemichordata : Harrimaniidae) from Barkley Sound. The Biological Bulletin. 2002;202: 182-191.

11. Worsaae K, Sterrer W, Kaul-Strehlow S, Hay-Schmidt A, Giribet G. An anatomical description of a miniaturized acorn worm (Hemichordata, Enteropneusta) with asexual reproduction by paratomy. Plos One. 2012;7(11). DOI: 10.1371/journal.pone.0048529.

12. Menon K. Enteropneusta from Madras, contains an account of *D. bournei.* Q J Microsc Sci. 1904;97: 6.

13. Rao K. Enteropneusta from the east coast of India, with a note on the probable course of distribution of *Ptychodera flava.* Proc Plant Sci. 1962;55(5): 224-232.

14. van der Horst C. West-Indische Enteropneusten. Bijdragen tot de Dierkunde. 1924; 23(1): 33-60.

15. Robinson V. Report on a new species of Enteropneust. Trans Zool Soc London. 1927; 22(3): 361-364.

16. Palomares M, Pauly D. SeaLifeBase Vancouver 2011 [cited 2014 May]. Available: http://www.sealifebase.org/.

17. Por F. Lessepsian Migration. The influx of Red Sea biota into the Mediterranean by way of the Suez Canal. In: Billings W, Golley F, Lange O, Olsen J, editors. Ecological Studies. Berlin: Springer-Verlag; 1978. p. 228.

18. Cevik C, Ergüden D. First Record for Two Species [*Balanoglossus clavigerus* delle Chiaje, 1829, *Glandiceps talaboti* (Marion, 1876)] of the Phylum Hemichordata on the coast of Turkey. Turk J Zool. 2005;29: 141-145.

19. Hinrichs H, Jacobi L. *Saccoglossus pygmaeus,* eine neue Enteropneustenart aus der südlichen Nordsee. Zool Anz. 1938;121: 25-32.

20. MarBEF. European node of the Ocean Biogeographic Information System.: Marine Biodiversity and Ecosystem Functioning EU Network of Excellence; 2004. European node of the Ocean Biogeographic Information System. [cited 2014 May]. Available: http://www.marbef.org/data/.

21. Cedhagen T, Hansson HG. Biology and distribution of hemichordates (Enteropneusta) with emphasis on Harrimaniidae and description of *Protoglossus bocki* sp nov from Scandinavia. Helgoland Mar Res. 2013;67(2): 251-265.

22. Giray C, King GM. *Protoglossus graveolens*, a new hemichordate (Hemichordata: Enteropneusta: Harrimaniidae) from the northwest Atlantic. Proc Biol Soc Wash. 1996;109: 430-445.

23. Caullery M, Mesnil F. Sur une nouvelle espece del *Balanoglossus* (*B. koehleri*) habitant les cotes de la Manche. Cr Soc Biol. 1900;52: 256-259.

24. Burdon-Jones C. Observations on the enteropneust, *Protoglossus koehleri* (Caullery & Mesnil). Proc Zool Soc London. 1956;127(1): 35-58.

25. Thomas I. *Saccoglossus apatensis*, a new species of enteropneust from South Australia. T Roy Soc South Aust. 1955;79: 167-176.

26. Thomas I. Two Species of *Saccoglossus* (Enteropneusta) from South Australia. T Roy Soc South Aust. 1968;92: 73-84.

27. King GM, Giray C, Kornfield I. A new hemichordate, *Saccoglossus bromophenolosus* (Hemichordata: Enteropneusta: Harrimaniidae), from North America. Proc Biol Soc Wash. 1994;107: 383-390.

28. Brambell F, Goodhart C. *Saccoglossus horsti*, sp. n., an enteropneust occurring in the Solent. J Mar Biol Assoc. 1941;25: 283-301.

29. Tchang S, Koo G. Two enteropneusts in Jiaozhou Bay. Publication of the Beijing Institute of Zoology. 1935;13: 1-12.

30. Kapelus F. The Anatomy of the Enteropneust *Saccoglossus inhacensis* sp. n. Annals of the Natal Museum. 1936;9(1): 37-94.

31. Hyman L. The Invertebrates 5: Smaller Coelomate Groups New York: McGraw-Hill; 1959.

32. Agassiz A. The history of *Balanoglossus* and *Tornaria.* Memoirs of the American Academy of Arts and Sciences. 1873;9(2): 421-436.

33. Rao K. Two species of Enteropneusta from off the coast of Madras. Proceedings of the Indian Science Congress. 1957;42: 301.

34. Wagner N. Die Wirbellosen des Weissen Meeres. Zoologische Forschungen an der Küste des Solowetzkischen Meerbusens in den Sommermonaten der Jahre 1877, 1878, 1879 und 1882. Leipzig: Engelmann; 1885.

35. Ezhova O, Malakhov V. Three-dimensional structure of the skeleton and buccal diverticulum of an acorn worm *Saccoglossus mereschkowskii* Wagner, 1885 (Hemichordata: Enteropneusta). Invertebr Zool. 2009;6(2): 103-116.

36. Benham W. Memoirs: *Balanoglossus otagoensis*, n. sp. Q J Microsc Sci. 1899;2(42): 497-504.

37. Benham W. On the Occurrence of *Balanoglossus*. Transactions and Proceeding of the New Zealand Institute. 1899;32: 9-10.

38. Gordon D, Cooper R, Campbell H. Phylum Hemichordata: acorn worms, pterobranchs, graptolites. In: Gordon D, editor. New Zealand inventory of biodiversity: 1 Kingdom Animalia: Radiata, Lophotrochozoa, Deuterostomia. 2009. p. 401-408.

39. Cameron CB, Deland C, Bullock TH. A revision of the genus *Saccoglossus* (Hemichordata: Enteropneusta:Harrimaniidae) with taxonomic descriptions of five new species from the Eastern Pacific. Zootaxa. 2010;2483: 1-22.

40. Ritter WE. The movements of the Enteropneusta and the mechanisms by which they are accomplished. Biol Bull. 1902;3: 255-261.

41. Tattersall W. Enteropneusta from the west coast of Ireland. Annual Report of Fisheries, Ireland Scientific Investigations. 1905;1902: 213-214.

42. Spengel J. Die Enteropneusten des Golfes von Neapel. Fauna und Flora des Golfes von Neapel und der angrenzenden Meeres-Abschnitte. Berlin: Herausgegeben von der Zoologischen Station zu Neapel.; 1893.

43. Woodwick K, Sesenbaugh T. *Saxipendium coronatum*, new genus, new species (Hemichordata: Enteropneusta): the unusual spaghetti worms of the Galápagos Rift hydrothermal vents. Proc Biol Soc Wash. 1985;98: 351-365.

44. Holland ND, Osborn KJ, Kuhnz LA. A new deep-sea species of harrimaniid enteropneust (Hemichordata). Proc Biol Soc Wash. 2012;125(3): 228-240.

45. Gilchrist J. *Xenopleura vivipara*, g. et sp. n. (Enteropneusta). Q J Microsc Sci. 1925;69: 555-573.

46. Punnett R. The Enteropneusta. In: Gardiner J, editor. The Fauna and Geography of the Maldive and Laccadive Archipelagos. London: Cambridge University Press; 1903. p. 631-679.

47. Girard C. The Committee on Mr. Girard's descriptions of new Nemerteans and Planarians, reported in favor of publication. P Acad Nat Sci Phila. 1853;6: 367.

48. Willey A. Enteropneusta from the South Pacific, with notes on the West Indian species. Willey's Zoological Results. 1899;3: 32-335.

49. Hill J. Preliminary note on a *Balanoglossus* from the coast of New South Wales. P Linn Soc N S W. 1894;2(8): 324.

50. Gibbs P. Macrofauna of the intertidal sand flats on low wooded islands, northern Great Barrier Reef. Philos Trans R Soc Lond B Biol Sci. 1978;284: 81-97.

51. Gilchrist J. New forms of the Hemichordata from South Africa. T Phil Soc S Afr. 1908;17: 151-76.

52. Muller F. Observações sobre a fauna marinha da costa de Sta. Catharina. Rev Mus Paulista. 1898;3: 31-40.

53. delle Chiaje S. Memorie sulla storia e notomia degli animali senza vertebre del Regno di Neapel. Napoli. 1829;4: 1-72.

54. Sawaya P. *Balanoglossus gigas* Fr. Müller rediscovered on the Brazilian Coast. Nature. 1951;167: 730-731.

55. Johnson AS, Hillestad HO, Shanholtzer SF, Shanholtzer GF. An ecological survey of the coastal region of Georgia. Scientific Monograph Series. Washington DC: National Park Services; 1974. p. 233.

56. van der Horst C. The Enteropneusta from Inyack Island, Delagoa Bay. Annals of the South African Museum. 1940;32: 293-380.

57. Macnae W, Kalk M. The fauna and flora of sand flats at Inhaca Island, Moçambique. J Anim Ecol. 1962;31(1): 93-124.

58. Kuwano H. On a new Enteropneust from Misaki, *Balanoglossus misakiensis* n. sp. Annot Zool Japon. 1902;4(2): 77-84.

59. Gilchrist J. On Two New Species of *Ptychodera* (*P. proliferans* and *P. natalensis*). Annals of the South African Museum. 1908;6: 207-212.

60. Day J. A Guide to Marine Life on South African Shores. Cape Town & Rotterdam: A. A. Balkema; 1969.

61. Ritter W. Movements of Enteropneusta. J Royal Mic Soc. 1902;25: 43.

62. Pillay T. On the occurrence of *Glossobalanus parvulus* (Punnett) on the Okhamandal (Kathiawar) coast. Curr Sci. 1950;19(5): 156.

63. Belichov D. Contributions to the Systematica of Enteropneusta. Proceedings of the 3rd Congress of the Russian Zoologists, Anatomists, and Histologists; 1928; Leningrad.

64. Dautov SS, Nezlin LP, Yushin VV. Structure of the digestive tract of tornaria larva in Enteropneusta (Hemichordata). Helgolander Meeresun. 1994;48: 107-121.

65. Giard A. Sur un type synthétique d'annélide (*Anoplonereis herrmanni*), commensal des *Balanoglossus.* Rev Int Sci Biol. 1882;10: 285-286.

66. Miyamoto M, Saito Y. Morphology and development of a new species of *Balanoglossus* (Hemichordata: Enteropneusta: Ptychoderidae) from Shimoda, Japan. Zool Sci. 2007;24(12): 1278-1285.

67. van der Horst C. On a new South African species of *Balanoglossus* and a comparison between it and *Balanoglossus capensis* (Gilchrist). Annals of the South African Museum. 1937;32: 69-93.

68. Cameron CB, Ostiguy A. Three new species of *Glossobalanus* (Hemichordata: Enteropneusta: Ptychoderidae) from western North America. Zootaxa. 2013;3630(1): 143-154.

69. Willey A. *Glossobalanus berkeleyi*, a new enteropneust from the West Coast. T Roy Soc Can. 1931;5: 19-28.

70. Seavy D. An Introduction to the biology of *Glossobalanus berkeleyi* in southern Puget Sound: University of Puget Sound; 1965.

71. Ditadi AS, Mendes EG, Bianconcini ES. Influence of body mass and environmental oxygen tension on the oxygen consumption rates of an enteropneust, *Glossobalanus crozieri*. Brazilian Journal of Medical and Biomedical Research. 1997;30(12): 1441-1444.

72. Björnberg T. On Enteropneusta from Brazil. PhD Thesis, Universidade de São Paulo. 1959. Available: http://www.scielo.br/pdf/bioce/v10n1/v10n1a01.pdf

73. Spengel J. Neue Beiträge zur Kenntnis der Enteropneustenart aus dem Golf von Neapel, nebst Beobachtungen über den postbranchialen Darm der Ptychoderiden. Zoologische Jahrbücher: Abteilung für Anatomie und Ontogenie dur Thiere. 1904;20: 315-362.

74. Hill J. XIV The Enteropneusta Part II. Australian Museum Memoir. 1897;3(5): 336-348.

75. Rao K. Tornaria from Madras (Enteropneusta). Hydrobiologia. 1955;7(3): 269-278.

76. Meek A. *Glossobalanus marginatus*, a new species of Enteropneusta from the North Sea. Q J Microsc Sci. 1922;66: 579-594.

77. WoRMS Editorial Board. World Register of Marine Species 2015 [cited 2014 May]. Available from: http://www.marinespecies.org/.

78. Kowalevsky A. Anatomie des *Balanoglossus*. Mem Acad Imp Sci St Petersburg. 1866;7(10): 16.

79. Willey A. On *Ptychodera flava*, Eschscholtz. Q J Microsc Sci. 1898;40: 165-184.

80. van der Horst C. On some Enteropneusta. Annals of the Transvaal Museum. 1932;14(4): 414-430.

81. Punnett R. The Enteropneusta. In: Gardiner J, editor. The Fauna and geography of the Maldive and Laccadive Archipelagos. III. London: Cambridge University Press; 1906. p. 641-680.

82. Tchiang S, Liang X. Descripition of a new species of Enteropneusta, *Glossobalanus polybranchioporus* from China seas. Acta Zool Sinica. 1965;2(1): 1-10.

83. van der Horst C. Observations on some Enteropneusta. Papers from Dr. Th. Mortensen's Pacific Expedition 1914-16. Vidensk Medd naturhist Foren København. 1930;87: 135-200.

84. Okuda S. The Enteropneusta from the Palau Islands. Journal of the Faculty of Science, Hokkaido University: Zoology. 1939;7: 17-25.

85. Koehler R. Contribution a l'etude des Enteropneustes. Recherch anat. sur le *Balanoglossus sarniensis* nov. sp. Internat Monats Anat Hist. 1886;3: 139-190.

86. Eschscholtz F. Bericht über die zoologische Ausbeute während der Reise von Kronstadt bis St. Peter und Paul. Oken's Isis. 1825;6: 733-747.

87. Dakin W. A new Species of Enteropneusta, *Ptychodera pelsarti*, from the Abrolhos Islands. Journal of the Linnean Society of London, Zoology. 1916;33(222): 85-100.

88. Kirsteuer E. *Ptychodera flava* (Enteropneust) von Tanikely, Madagaskar der Österreichischen ergebnisse Indo-Westpazifik-Expedition 1959/60. Zool Anz. 1965;175: 371-377.

89. Uribe M, Larrain A. Estudios biologicos en el enterepneusto *Ptychodera flava* Eschscholtz, 1825 de Bahia Concepcion, Chile. I: Aspectos morfologicos y ecologicos. Gayana Zool. 1992;56(3-4): 141-180.

90. Spengel J. Studien über die enteropneusten der Siboga-expedition nebst beobachtungen an verwandten arten.: University of California Libraries; 1907.

91. Marion A. Sur deux especes de *Balanoglossus*. CR de l'Institut. 1885;101: 1289-1291.

92. Burdon-Jones C, Kott P, Richardson B. Zoological Catalogue of Australia Volume 34: Hemichordata, Tunicata, Cephalochordata: CSIRO Publishing; 1998.

93. An J, Li X. First record of the family Spengeliidae (Hemichordata: Enteropneusta) from Chinese waters, with description of a new species. J Nat Hist. 2005;39(22): 1995-2004.

94. Marion A. Dragages profondes au large de Marseille, note preliminaire. Rev Sci Nat. 1876;4(4): 469.

95. Cameron CB, Perez M. Spengelidae (Hemichordata: Enteropneusta) from the Eastern Pacific including a new species, *Schizocardium californicum*, from California. Zootaxa. 2012(3569): 79-88.

96. Willey A. *Spengelia*, a new genus of Enteropneusta. Q J Microsc Sci. 1898;40: 623-630.

97. Petersen J. Contribuição para o conhecimento da ecologia e da fisiologia de Enteropneustos do Brasil com descrição de uma nova espécie, *Willeyia loya* Sp.n., *Tese para Doutoramento em Ciências.*: Universidade de São Paulo, Brasil; 1965.

98. Holland ND, Kuhnz LA, Osborn KJ. Morphology of a new deep-sea acorn worm (class Enteropneusta, phylum Hemichordata): A part-time demersal drifter with externalized ovaries. J Morphol. 2012;273(7): 661-671.

99. Priede IG, Osborn KJ, Gebruk AV, Jones D, Shale D, Rogacheva A, et al. Observations on torquaratorid acorn worms (Hemichordata, Enteropneusta) from the North Atlantic with descriptions of a new genus and three new species. Invertebr Biol. 2012;131(3): 244-257.

100. Osborn KJ, Gebruk AV, Rogacheva A, Holland ND. An Externally Brooding Acorn Worm (Hemichordata, Enteropneusta, Torquaratoridae) from the Russian Arctic. Biol Bull. 2013;225(2): 113-123.

101. Holland ND, Jones WJ, Ellena J, Ruhl HA, Smith KL. A new deep-sea species of epibenthic acorn worm (Hemichordata, Enteropneusta). Zoosystema. 2009;31(2): 333-346.

102. Holland ND, Clague DA, Gordon DP, Gebruk A, Pawson DL, Vecchione M. 'Lophenteropneust' hypothesis refuted by collection and photos of new deep-sea hemichordates. Nature. 2005;434(7031): 374-376.

103. Harmer S, Ridewood W. The Pterobranchia of the Scottish National Antarctic Expedition (1902-1904). T Roy Soc Edin. 1913;49(7): 531-565.

104. Bayer F. A new species of *Cephalodiscus* (Hemichordata: Pterobranchia), the first record from the tropical Western Atlantic. Bulletin of Marine Science Gulf Carribean. 1962;12: 306-312.

105. Johnston T, Muirhead N. *Cephalodiscus*. Report of the British Australian and New Zealand Antarctic Expedition. 1951. p. 91-120.

106. Emig C. On a new species of *Cephalodiscus, C. caliciformis* sp. nov. (Hemichordata, Pterobranchia), collected off Madagascar. Bulletin du Museum National d'Historie Naturelle, France 3E, Zool. 1977;493: 1077-1083.

107. Andersson K. Die Pterobranchier der Schwedischen Sudpolar-Expedition, 1901-1903. Scientific Results of the Swedish Sudpolar Expedition. 1907;5: 1-122.

108. Markham J. The Species of *Cephalodiscus* Collected During Operation Deep Freeze, 1956-1959. In: Llano G, Wallen I, editors. Biology of the Antarctic Seas IV: American Geophysical Union; 1971. p. 83-110.

109. Schiaparelli S, Cattaneo-Vietti R, Mierzejewski P. A "protective shell" around the larval cocoon of *Cephalodiscus densus* Andersson, 1907 (Graptolithoidea, Hemichordata). Polar Biol. 2004;27(12): 813-817.

110. M'Intosh W. Preliminary notice of *Cephalodiscus*, a new type allied to Prof. Allman's *Rhabdopleura* dredged in *H.M.S. 'Challenger.'*. Annals and Magazine of Natural History. 1882;10: 337-348.

111. M'Intosh W. Report on *Cephalodiscus dodecalophus* M'Intosh, a new type of Polyzoa, procured on the voyage of *H.M.S. Challenger* during the years 1873-76. In: Thompson C, Murray J, editors. Challenger Reports. 20. Edinburgh: Neill; 1887. p. 1-37.

112. Ridewood W. *Cephalodiscus* of the "Terra Nova" Expedition, 1910. British Antarctic ("Terra Nova") Expedition Natural History Report: Zoology. British Museum of Natural History; 1918; 4: 11-82.

113. John C. *Cephalodiscus*. Discovery Reports. 1931;3:223-260.

114. Ridewood W. A new species of *Cephalodiscus* (*C. gilchristi*) from the Cape Seas. 1908;4: 173-192.

115. Flessner T, Jautelat R, Scholz U, Winterfeldt E. Cephalostatin Analogoues - Synthesis and Biological Activity. In: Herz W, Falk H, Kirby G, editors. Fortschritte der Chemie organischer Naturstoffe Progress in the Chemistry of Organic Natural Products. 87. New York: Springer; 2004. p. 1-80.

116. Pettit G, Inoue M, Kamano Y, Herald D, Arm C, Dufrense C, et al. Isolation and structure of the powerful cell growth inhibitor cephalostatin 1. J Am Chem Soc. 1988;110(6): 2006-2007.

117. Harmer S. The Pterobranchia of the Siboga Expedition. Siboga Expedition Monograph. 1905. p. 1-31.

118. Dilly P. The habitat and behaviour of *Cephalodiscus gracilis* (Pterobranchia, Hemichordata) from Bermuda. J Zool. 1985;207(2): 223-239.

119. Dilly P. *Cephalodiscus graptolitoides* sp. nov. a probable extant graptolite. J Zool. 1993;229(1): 69-78.

120. Ridewood W. Pterobranchia; *Cephalodiscus*. National Antarctic Expedition "Discovery" Natural History. 1907;2: 1-67.

121. Urbanek A, Zielinski K. Preliminary Report on *Cephalodiscus* (Pterobranchia) from Admiralty Bay, King George Island, South Shetland Islands, West Antarctica. Bulletin of the Polish Academy of Sciences. 1982;29: 257-262.

122. Schepotieff A. Die Pterobranchier des Indischen Ozeans. Zoologische Jahrbuecher Abteilung fuer Systematik Oekologie und Geographie der Tiere. 1909;28: 429-445.

123. John C. On the development of *Cephalodiscus*. Discovery Reports. 1932;6: 191-204.

124. Lankester E. On a new species of *Cephalodiscus* (*C. nigrescens*) from the Antarctic Ocean. P R Soc London. 1905;76B: 400-402.

125. Andersson K. Die Pterobranchier der Schwedischen Sudpolar-Expedition, 1901-1903. Wissenschaftliche Ergebnisse der Schwedischen Sudpolar-Expedition. Stockholm, Sweden; 1907. p. 1-122

126. Norman J. *Rhabdopleura.* British Antarctic ("Terra Nova") Expedition Natural History Report: Zoology. British Museum of Natural History; 1921. p. 95-102.

127. Brownsey P, Baker A, editors. The New Zealand Biota: What do we know after 200 years? Systematics Association of New Zealand. Victoria University, Wellington: National Museum of New Zealand. 1983

128. Hincks T. A History of the British Marine Polyzoa. London: Voorst; 1880.

129. Sato A. Seasonal reproductive activity in the pterobranch hemichordate *Rhabdopleura compacta*. J Mar Biol Assoc UK. 2008;88: 1033-1041.

130. Jullien J. Description d'un Bryozoaire nouveau du genre *Rhabdopleura*. B Soc Zool Fr. 1890;15: 180-183.

131. Palaeoecology and biostratigraphy of graptolites. 2nd International Conference of the Graptolite Working Group of the International Palaeontological Association. Cambridge University: Blackwell Scientific. 1981.

132. Allman G. *Rhabdopleura normani*, Allman, nov. gen. et sp. Report of the British Association for the Advancement of Science. 1869(1868): 311-312.

133. Dilly P, Ryland J. An intertidal *Rhabdopleura* (Hemichordata, Pterobranchia) from Fiji. J Zool. 1985;205(4): 611-623.
