# Supplementary material for: The Global Diversity of Hemichordata
Source: PLoS One. 2016 Oct 4;11(10):e0162564. doi: 10.1371/journal.pone.0162564 (PMC5049775; doi:10.1371/journal.pone.0162564)
Supplement: S2 Table — (PDF) [file pone.0162564.s002.pdf]

S2 Table. Extant Hemichordate Species and Geographic Regions They Inhabit. Reports of species locales were compiled from monographs, books, government reports and published literature (see Methods for details). Reports were verified and initially binned according to marine provinces based on Spalding et al. 2007 [1]. Some regions were then consolidated for broader regional impact. In addition to the total number of species found in each geographic region, the numbers of enteropneust and pterobranch species are also given. See Fig 6 for a visual representation of this data and S2 Table for more marine province data.

Geographic Region	Total Species	Enteropneusts	Pterobranchs
**Agulhas**	6	5	1
**Arctic**	5	4	1
**Bay of Bengal & Andaman**	9	9	0
**Central Indian Ocean Islands**	10	10	0
**Cold Temperate NE Pacific**	16	16	0
**Cold Temperate NW Atlantic**	5	5	0
**Cold Temperate NW Pacific**	10	9	1
**Coral Triangle and Sahul Shelf**	8	6	2
**Deep Sea North Atlantic**	3	3	0
**E/SE Australian Shelf**	4	2	2
**Eastern Indo-Pacific Islands**	1	1	0
**Gulf of Guinea**	1	1	0
**Java Transitional**	1	1	0
**Lusitanian**	5	4	1
**Magellanic**	4	0	4
**Mediterranean Sea**	6	5	1
**NE Australian Shelf**	6	6	0
**New Zealand**	5	3	2
**Northern European Seas**	12	10	2
**Red Sea**	2	2	0
**Scotia Sea**	6	0	6
**South China Sea**	1	1	0
**Tropical Eastern Pacific**	1	1	0
**Tropical NW Atlantic**	10	7	3
**Tropical NW Pacific**	2	2	0
**Tropical SW Atlantic**	1	1	0
**Tropical SW Pacific**	5	3	2
**W/SW Australian Shelf**	5	5	0
**Warm Temperate NE Pacific**	10	10	0
**Warm Temperate NW Atlantic**	4	4	0
**Warm Temperate NW Pacific**	7	7	1
**Warm Temperate SE Pacific**	2	2	0
**Warm Temperate SW Atlantic**	6	6	0
**West & South Indian Shelf**	6	4	2
**Western Indian Ocean**	11	10	1

References

1. Spalding MD, Fox HE, Allen GR, Davidson N, Ferdaña ZA, Finlayson M, et al. Marine ecoregions of the world: A bioregionalization of coastal and shelf areas. BioScience. 2007;57(7): 573-583.

2. Okuda S, Yamada M. Enteropneusta of Akkeshi Bay. Publications of the Akkeshi Marine Biological Station. 1955;6: 1-7.

3. von Willemoes-Suhm R. Biologische Beobachtungen über niedere Meeresthiere. Ueber *Balanoglossus kupfferi* aus den Oeresund. Z Wiss Zool. 1871;21: 380-396.

4. GBIF. Global Biodiversity Information Facility. 2011. Ver. 1.3.1 [cited 2014 May] Available: http://www.gbif.org/.

5. Cunningham J. Tornaria and Actinotropha of the British Coasts. Nature. 1886;34(877): 361.

6. International Oceanographic Commission of UNESCO. The Ocean Biogeographic Information System. 2007 [cited 2014 May]. Available: http://iobis.org/.

7. Hansson H. NEAT (North East Atlantic Taxa): South Scandinavian marine "Aschelminth" (excl. Nemetoda 1997 [cited 2014 May]. Available: http://www.tmbl.gu.se.

8. Ritter W. *Harrimania maculosa*, a new genus and species of Enteropneusta from Alaska, with special regard to the charcter of its notochord. Proc Wash Acad Sci. 1900;2: 111-132.

9. Deland C, Cameron C, Rao K, Ritter W, Bullock T. A taxonomic revision of the family Harrimaniidae (Hemichordata: Enteropneusta) with descriptions of seven species from the Eastern Pacific. Zootaxa. 2010: 1-30.

10. Cameron CB. The anatomy, life habits, and later development of a new species of enteropneust, *Harrimania planktophilus* (Hemichordata : Harrimaniidae) from Barkley Sound. The Biological Bulletin. 2002;202: 182-191.

11. Worsaae K, Sterrer W, Kaul-Strehlow S, Hay-Schmidt A, Giribet G. An anatomical description of a miniaturized acorn worm (Hemichordata, Enteropneusta) with asexual reproduction by paratomy. Plos One. 2012;7(11). DOI: 10.1371/journal.pone.0048529.

12. Menon K. Enteropneusta from Madras, contains an account of *D. bournei.* Q J Microsc Sci. 1904;97: 6.

13. Rao K. Enteropneusta from the east coast of India, with a note on the probable course of distribution of *Ptychodera flava.* Proc Plant Sci. 1962;55(5): 224-232.

14. van der Horst C. West-Indische Enteropneusten. Bijdragen tot de Dierkunde. 1924; 23(1): 33-60.

15. Robinson V. Report on a new species of Enteropneust. Trans Zool Soc London. 1927; 22(3): 361-364.

16. Palomares M, Pauly D. SeaLifeBase Vancouver 2011 [cited 2014 May]. Available: http://www.sealifebase.org/.

17. Por F. Lessepsian Migration. The influx of Red Sea biota into the Mediterranean by way of the Suez Canal. In: Billings W, Golley F, Lange O, Olsen J, editors. Ecological Studies. Berlin: Springer-Verlag; 1978. p. 228.

18. Cevik C, Ergüden D. First Record for Two Species [*Balanoglossus clavigerus* delle Chiaje, 1829, *Glandiceps talaboti* (Marion, 1876)] of the Phylum Hemichordata on the coast of Turkey. Turk J Zool. 2005;29: 141-145.

19. Hinrichs H, Jacobi L. *Saccoglossus pygmaeus,* eine neue Enteropneustenart aus der südlichen Nordsee. Zool Anz. 1938;121: 25-32.

20. MarBEF. European node of the Ocean Biogeographic Information System.: Marine Biodiversity and Ecosystem Functioning EU Network of Excellence; 2004. European node of the Ocean Biogeographic Information System. [cited 2014 May]. Available: http://www.marbef.org/data/.

21. Cedhagen T, Hansson HG. Biology and distribution of hemichordates (Enteropneusta) with emphasis on Harrimaniidae and description of *Protoglossus bocki* sp nov from Scandinavia. Helgoland Mar Res. 2013;67(2): 251-265.

22. Giray C, King GM. *Protoglossus graveolens*, a new hemichordate (Hemichordata: Enteropneusta: Harrimaniidae) from the northwest Atlantic. Proc Biol Soc Wash. 1996;109: 430-445.

23. Caullery M, Mesnil F. Sur une nouvelle espece del *Balanoglossus* (*B. koehleri*) habitant les cotes de la Manche. Cr Soc Biol. 1900;52: 256-259.

24. Burdon-Jones C. Observations on the enteropneust, *Protoglossus koehleri* (Caullery & Mesnil). Proc Zool Soc London. 1956;127(1): 35-58.

25. Thomas I. *Saccoglossus apatensis*, a new species of enteropneust from South Australia. T Roy Soc South Aust. 1955;79: 167-176.

26. Thomas I. Two Species of *Saccoglossus* (Enteropneusta) from South Australia. T Roy Soc South Aust. 1968;92: 73-84.

27. King GM, Giray C, Kornfield I. A new hemichordate, *Saccoglossus bromophenolosus* (Hemichordata: Enteropneusta: Harrimaniidae), from North America. Proc Biol Soc Wash. 1994;107: 383-390.

28. Brambell F, Goodhart C. *Saccoglossus horsti*, sp. n., an enteropneust occurring in the Solent. J Mar Biol Assoc. 1941;25: 283-301.

29. Tchang S, Koo G. Two enteropneusts in Jiaozhou Bay. Publication of the Beijing Institute of Zoology. 1935;13: 1-12.

30. Kapelus F. The Anatomy of the Enteropneust *Saccoglossus inhacensis* sp. n. Annals of the Natal Museum. 1936;9(1): 37-94.

31. Hyman L. The Invertebrates 5: Smaller Coelomate Groups New York: McGraw-Hill; 1959.

32. Agassiz A. The history of *Balanoglossus* and *Tornaria.* Memoirs of the American Academy of Arts and Sciences. 1873;9(2): 421-436.

33. Rao K. Two species of Enteropneusta from off the coast of Madras. Proceedings of the Indian Science Congress. 1957;42: 301.

34. Wagner N. Die Wirbellosen des Weissen Meeres. Zoologische Forschungen an der Küste des Solowetzkischen Meerbusens in den Sommermonaten der Jahre 1877, 1878, 1879 und 1882. Leipzig: Engelmann; 1885.

35. Ezhova O, Malakhov V. Three-dimensional structure of the skeleton and buccal diverticulum of an acorn worm *Saccoglossus mereschkowskii* Wagner, 1885 (Hemichordata: Enteropneusta). Invertebr Zool. 2009;6(2): 103-116.

36. Benham W. Memoirs: *Balanoglossus otagoensis*, n. sp. Q J Microsc Sci. 1899;2(42): 497-504.

37. Benham W. On the Occurrence of *Balanoglossus*. Transactions and Proceeding of the New Zealand Institute. 1899;32: 9-10.

38. Gordon D, Cooper R, Campbell H. Phylum Hemichordata: acorn worms, pterobranchs, graptolites. In: Gordon D, editor. New Zealand inventory of biodiversity: 1 Kingdom Animalia: Radiata, Lophotrochozoa, Deuterostomia. 2009. p. 401-408.

39. Cameron CB, Deland C, Bullock TH. A revision of the genus *Saccoglossus* (Hemichordata: Enteropneusta:Harrimaniidae) with taxonomic descriptions of five new species from the Eastern Pacific. Zootaxa. 2010;2483: 1-22.

40. Ritter WE. The movements of the Enteropneusta and the mechanisms by which they are accomplished. Biol Bull. 1902;3: 255-261.

41. Tattersall W. Enteropneusta from the west coast of Ireland. Annual Report of Fisheries, Ireland Scientific Investigations. 1905;1902: 213-214.

42. Spengel J. Die Enteropneusten des Golfes von Neapel. Fauna und Flora des Golfes von Neapel und der angrenzenden Meeres-Abschnitte. Berlin: Herausgegeben von der Zoologischen Station zu Neapel.; 1893.

43. Woodwick K, Sesenbaugh T. *Saxipendium coronatum*, new genus, new species (Hemichordata: Enteropneusta): the unusual spaghetti worms of the Galápagos Rift hydrothermal vents. Proc Biol Soc Wash. 1985;98: 351-365.

44. Holland ND, Osborn KJ, Kuhnz LA. A new deep-sea species of harrimaniid enteropneust (Hemichordata). Proc Biol Soc Wash. 2012;125(3): 228-240.

45. Gilchrist J. *Xenopleura vivipara*, g. et sp. n. (Enteropneusta). Q J Microsc Sci. 1925;69: 555-573.

46. Punnett R. The Enteropneusta. In: Gardiner J, editor. The Fauna and Geography of the Maldive and Laccadive Archipelagos. London: Cambridge University Press; 1903. p. 631-679.

47. Girard C. The Committee on Mr. Girard's descriptions of new Nemerteans and Planarians, reported in favor of publication. P Acad Nat Sci Phila. 1853;6: 367.

48. Willey A. Enteropneusta from the South Pacific, with notes on the West Indian species. Willey's Zoological Results. 1899;3: 32-335.

49. Hill J. Preliminary note on a *Balanoglossus* from the coast of New South Wales. P Linn Soc N S W. 1894;2(8): 324.

50. Gibbs P. Macrofauna of the intertidal sand flats on low wooded islands, northern Great Barrier Reef. Philos Trans R Soc Lond B Biol Sci. 1978;284: 81-97.

51. Gilchrist J. New forms of the Hemichordata from South Africa. T Phil Soc S Afr. 1908;17: 151-76.

52. Muller F. Observações sobre a fauna marinha da costa de Sta. Catharina. Rev Mus Paulista. 1898;3: 31-40.

53. delle Chiaje S. Memorie sulla storia e notomia degli animali senza vertebre del Regno di Neapel. Napoli. 1829;4: 1-72.

54. Sawaya P. *Balanoglossus gigas* Fr. Müller rediscovered on the Brazilian Coast. Nature. 1951;167: 730-731.

55. Johnson AS, Hillestad HO, Shanholtzer SF, Shanholtzer GF. An ecological survey of the coastal region of Georgia. Scientific Monograph Series. Washington DC: National Park Services; 1974. p. 233.

56. van der Horst C. The Enteropneusta from Inyack Island, Delagoa Bay. Annals of the South African Museum. 1940;32: 293-380.

57. Macnae W, Kalk M. The fauna and flora of sand flats at Inhaca Island, Moçambique. J Anim Ecol. 1962;31(1): 93-124.

58. Kuwano H. On a new Enteropneust from Misaki, *Balanoglossus misakiensis* n. sp. Annot Zool Japon. 1902;4(2): 77-84.

59. Gilchrist J. On Two New Species of *Ptychodera* (*P. proliferans* and *P. natalensis*). Annals of the South African Museum. 1908;6: 207-212.

60. Day J. A Guide to Marine Life on South African Shores. Cape Town & Rotterdam: A. A. Balkema; 1969.

61. Ritter W. Movements of Enteropneusta. J Royal Mic Soc. 1902;25: 43.

62. Pillay T. On the occurrence of *Glossobalanus parvulus* (Punnett) on the Okhamandal (Kathiawar) coast. Curr Sci. 1950;19(5): 156.

63. Belichov D. Contributions to the Systematica of Enteropneusta. Proceedings of the 3rd Congress of the Russian Zoologists, Anatomists, and Histologists; 1928; Leningrad.

64. Dautov SS, Nezlin LP, Yushin VV. Structure of the digestive tract of tornaria larva in Enteropneusta (Hemichordata). Helgolander Meeresun. 1994;48: 107-121.

65. Giard A. Sur un type synthétique d'annélide (*Anoplonereis herrmanni*), commensal des *Balanoglossus.* Rev Int Sci Biol. 1882;10: 285-286.

66. Miyamoto M, Saito Y. Morphology and development of a new species of *Balanoglossus* (Hemichordata: Enteropneusta: Ptychoderidae) from Shimoda, Japan. Zool Sci. 2007;24(12): 1278-1285.

67. van der Horst C. On a new South African species of *Balanoglossus* and a comparison between it and *Balanoglossus capensis* (Gilchrist). Annals of the South African Museum. 1937;32: 69-93.

68. Cameron CB, Ostiguy A. Three new species of *Glossobalanus* (Hemichordata: Enteropneusta: Ptychoderidae) from western North America. Zootaxa. 2013;3630(1): 143-154.

69. Willey A. *Glossobalanus berkeleyi*, a new enteropneust from the West Coast. T Roy Soc Can. 1931;5: 19-28.

70. Seavy D. An Introduction to the biology of *Glossobalanus berkeleyi* in southern Puget Sound: University of Puget Sound; 1965.

71. Ditadi AS, Mendes EG, Bianconcini ES. Influence of body mass and environmental oxygen tension on the oxygen consumption rates of an enteropneust, *Glossobalanus crozieri*. Brazilian Journal of Medical and Biomedical Research. 1997;30(12): 1441-1444.

72. Björnberg T. On Enteropneusta from Brazil. PhD Thesis, Universidade de São Paulo. 1959. Available: http://www.scielo.br/pdf/bioce/v10n1/v10n1a01.pdf

73. Spengel J. Neue Beiträge zur Kenntnis der Enteropneustenart aus dem Golf von Neapel, nebst Beobachtungen über den postbranchialen Darm der Ptychoderiden. Zoologische Jahrbücher: Abteilung für Anatomie und Ontogenie dur Thiere. 1904;20: 315-362.

74. Hill J. XIV The Enteropneusta Part II. Australian Museum Memoir. 1897;3(5): 336-348.

75. Rao K. Tornaria from Madras (Enteropneusta). Hydrobiologia. 1955;7(3): 269-278.

76. Meek A. *Glossobalanus marginatus*, a new species of Enteropneusta from the North Sea. Q J Microsc Sci. 1922;66: 579-594.

77. WoRMS Editorial Board. World Register of Marine Species 2015 [cited 2014 May]. Available from: http://www.marinespecies.org/.

78. Kowalevsky A. Anatomie des *Balanoglossus*. Mem Acad Imp Sci St Petersburg. 1866;7(10): 16.

79. Willey A. On *Ptychodera flava*, Eschscholtz. Q J Microsc Sci. 1898;40: 165-184.

80. van der Horst C. On some Enteropneusta. Annals of the Transvaal Museum. 1932;14(4): 414-430.

81. Punnett R. The Enteropneusta. In: Gardiner J, editor. The Fauna and geography of the Maldive and Laccadive Archipelagos. III. London: Cambridge University Press; 1906. p. 641-680.

82. Tchiang S, Liang X. Descripition of a new species of Enteropneusta, *Glossobalanus polybranchioporus* from China seas. Acta Zool Sinica. 1965;2(1): 1-10.

83. van der Horst C. Observations on some Enteropneusta. Papers from Dr. Th. Mortensen's Pacific Expedition 1914-16. Vidensk Medd naturhist Foren København. 1930;87: 135-200.

84. Okuda S. The Enteropneusta from the Palau Islands. Journal of the Faculty of Science, Hokkaido University: Zoology. 1939;7: 17-25.

85. Koehler R. Contribution a l'etude des Enteropneustes. Recherch anat. sur le *Balanoglossus sarniensis* nov. sp. Internat Monats Anat Hist. 1886;3: 139-190.

86. Eschscholtz F. Bericht über die zoologische Ausbeute während der Reise von Kronstadt bis St. Peter und Paul. Oken's Isis. 1825;6: 733-747.

87. Dakin W. A new Species of Enteropneusta, *Ptychodera pelsarti*, from the Abrolhos Islands. Journal of the Linnean Society of London, Zoology. 1916;33(222): 85-100.

88. Kirsteuer E. *Ptychodera flava* (Enteropneust) von Tanikely, Madagaskar der Österreichischen ergebnisse Indo-Westpazifik-Expedition 1959/60. Zool Anz. 1965;175: 371-377.

89. Uribe M, Larrain A. Estudios biologicos en el enterepneusto *Ptychodera flava* Eschscholtz, 1825 de Bahia Concepcion, Chile. I: Aspectos morfologicos y ecologicos. Gayana Zool. 1992;56(3-4): 141-180.

90. Spengel J. Studien über die enteropneusten der Siboga-expedition nebst beobachtungen an verwandten arten.: University of California Libraries; 1907.

91. Marion A. Sur deux especes de *Balanoglossus*. CR de l'Institut. 1885;101: 1289-1291.

92. Burdon-Jones C, Kott P, Richardson B. Zoological Catalogue of Australia Volume 34: Hemichordata, Tunicata, Cephalochordata: CSIRO Publishing; 1998.

93. An J, Li X. First record of the family Spengeliidae (Hemichordata: Enteropneusta) from Chinese waters, with description of a new species. J Nat Hist. 2005;39(22): 1995-2004.

94. Marion A. Dragages profondes au large de Marseille, note preliminaire. Rev Sci Nat. 1876;4(4): 469.

95. Cameron CB, Perez M. Spengelidae (Hemichordata: Enteropneusta) from the Eastern Pacific including a new species, *Schizocardium californicum*, from California. Zootaxa. 2012(3569): 79-88.

96. Willey A. *Spengelia*, a new genus of Enteropneusta. Q J Microsc Sci. 1898;40: 623-630.

97. Petersen J. Contribuição para o conhecimento da ecologia e da fisiologia de Enteropneustos do Brasil com descrição de uma nova espécie, *Willeyia loya* Sp.n., *Tese para Doutoramento em Ciências.*: Universidade de São Paulo, Brasil; 1965.

98. Holland ND, Kuhnz LA, Osborn KJ. Morphology of a new deep-sea acorn worm (class Enteropneusta, phylum Hemichordata): A part-time demersal drifter with externalized ovaries. J Morphol. 2012;273(7): 661-671.

99. Priede IG, Osborn KJ, Gebruk AV, Jones D, Shale D, Rogacheva A, et al. Observations on torquaratorid acorn worms (Hemichordata, Enteropneusta) from the North Atlantic with descriptions of a new genus and three new species. Invertebr Biol. 2012;131(3): 244-257.

100. Osborn KJ, Gebruk AV, Rogacheva A, Holland ND. An Externally Brooding Acorn Worm (Hemichordata, Enteropneusta, Torquaratoridae) from the Russian Arctic. Biol Bull. 2013;225(2): 113-123.

101. Holland ND, Jones WJ, Ellena J, Ruhl HA, Smith KL. A new deep-sea species of epibenthic acorn worm (Hemichordata, Enteropneusta). Zoosystema. 2009;31(2): 333-346.

102. Holland ND, Clague DA, Gordon DP, Gebruk A, Pawson DL, Vecchione M. 'Lophenteropneust' hypothesis refuted by collection and photos of new deep-sea hemichordates. Nature. 2005;434(7031): 374-376.

103. Harmer S, Ridewood W. The Pterobranchia of the Scottish National Antarctic Expedition (1902-1904). T Roy Soc Edin. 1913;49(7): 531-565.

104. Bayer F. A new species of *Cephalodiscus* (Hemichordata: Pterobranchia), the first record from the tropical Western Atlantic. Bulletin of Marine Science Gulf Carribean. 1962;12: 306-312.

105. Johnston T, Muirhead N. *Cephalodiscus*. Report of the British Australian and New Zealand Antarctic Expedition. 1951. p. 91-120.

106. Emig C. On a new species of *Cephalodiscus, C. caliciformis* sp. nov. (Hemichordata, Pterobranchia), collected off Madagascar. Bulletin du Museum National d'Historie Naturelle, France 3E, Zool. 1977;493: 1077-1083.

107. Andersson K. Die Pterobranchier der Schwedischen Sudpolar-Expedition, 1901-1903. Scientific Results of the Swedish Sudpolar Expedition. 1907;5: 1-122.

108. Markham J. The Species of *Cephalodiscus* Collected During Operation Deep Freeze, 1956-1959. In: Llano G, Wallen I, editors. Biology of the Antarctic Seas IV: American Geophysical Union; 1971. p. 83-110.

109. Schiaparelli S, Cattaneo-Vietti R, Mierzejewski P. A “protective shell” around the larval cocoon of *Cephalodiscus densus* Andersson, 1907 (Graptolithoidea, Hemichordata). Polar Biol. 2004;27(12): 813-817.

110. M'Intosh W. Preliminary notice of *Cephalodiscus*, a new type allied to Prof. Allman's *Rhabdopleura* dredged in *H.M.S. 'Challenger.'*. Annals and Magazine of Natural History. 1882;10: 337-348.

111. M'Intosh W. Report on *Cephalodiscus dodecalophus* M'Intosh, a new type of Polyzoa, procured on the voyage of *H.M.S. Challenger* during the years 1873-76. In: Thompson C, Murray J, editors. Challenger Reports. 20. Edinburgh: Neill; 1887. p. 1-37.

112. Ridewood W. *Cephalodiscus* of the "Terra Nova" Expedition, 1910. British Antarctic ("Terra Nova") Expedition Natural History Report: Zoology. British Museum of Natural History; 1918; 4: 11-82.

113. John C. *Cephalodiscus*. Discovery Reports. 1931;3:223-260.

114. Ridewood W. A new species of *Cephalodiscus* (*C. gilchristi*) from the Cape Seas. 1908;4: 173-192.

115. Flessner T, Jautelat R, Scholz U, Winterfeldt E. Cephalostatin Analogoues - Synthesis and Biological Activity. In: Herz W, Falk H, Kirby G, editors. Fortschritte der Chemie organischer Naturstoffe Progress in the Chemistry of Organic Natural Products. 87. New York: Springer; 2004. p. 1-80.

116. Pettit G, Inoue M, Kamano Y, Herald D, Arm C, Dufrense C, et al. Isolation and structure of the powerful cell growth inhibitor cephalostatin 1. J Am Chem Soc. 1988;110(6): 2006-2007.

117. Harmer S. The Pterobranchia of the Siboga Expedition. Siboga Expedition Monograph. 1905. p. 1-31.

118. Dilly P. The habitat and behaviour of *Cephalodiscus gracilis* (Pterobranchia, Hemichordata) from Bermuda. J Zool. 1985;207(2): 223-239.

119. Dilly P. *Cephalodiscus graptolitoides* sp. nov. a probable extant graptolite. J Zool. 1993;229(1): 69-78.

120. Ridewood W. Pterobranchia; *Cephalodiscus*. National Antarctic Expedition "Discovery" Natural History. 1907;2: 1-67.

121. Urbanek A, Zielinski K. Preliminary Report on *Cephalodiscus* (Pterobranchia) from Admiralty Bay, King George Island, South Shetland Islands, West Antarctica. Bulletin of the Polish Academy of Sciences. 1982;29: 257-262.

122. Schepotieff A. Die Pterobranchier des Indischen Ozeans. Zoologische Jahrbuecher Abteilung fuer Systematik Oekologie und Geographie der Tiere. 1909;28: 429-445.

123. John C. On the development of *Cephalodiscus*. Discovery Reports. 1932;6: 191-204.

124. Lankester E. On a new species of *Cephalodiscus* (*C. nigrescens*) from the Antarctic Ocean. P R Soc London. 1905;76B: 400-402.

125. Andersson K. Die Pterobranchier der Schwedischen Sudpolar-Expedition, 1901-1903. Wissenschaftliche Ergebnisse der Schwedischen Sudpolar-Expedition. Stockholm, Sweden; 1907. p. 1-122

126. Norman J. *Rhabdopleura.* British Antarctic ("Terra Nova") Expedition Natural History Report: Zoology. British Museum of Natural History; 1921. p. 95-102.

127. Brownsey P, Baker A, editors. The New Zealand Biota: What do we know after 200 years? Systematics Association of New Zealand. Victoria University, Wellington: National Museum of New Zealand. 1983

128. Hincks T. A History of the British Marine Polyzoa. London: Voorst; 1880.

129. Sato A. Seasonal reproductive activity in the pterobranch hemichordate *Rhabdopleura compacta*. J Mar Biol Assoc UK. 2008;88: 1033-1041.

130. Jullien J. Description d'un Bryozoaire nouveau du genre *Rhabdopleura*. B Soc Zool Fr. 1890;15: 180-183.

131. Palaeoecology and biostratigraphy of graptolites. 2nd International Conference of the Graptolite Working Group of the International Palaeontological Association. Cambridge University: Blackwell Scientific. 1981.

132. Allman G. *Rhabdopleura normani*, Allman, nov. gen. et sp. Report of the British Association for the Advancement of Science. 1869(1868): 311-312.

133. Dilly P, Ryland J. An intertidal *Rhabdopleura* (Hemichordata, Pterobranchia) from Fiji. J Zool. 1985;205(4): 611-623.
